# Structure-Driven Feature Modeling for Robust Fish Detection in Complex Underwater Environments

**DOI:** 10.3390/ani16162519

**Published:** 2026-08-12

**Authors:** Yuting Wang, Ziyang Qi, Xiaojun Zhang, Qieqi Qian, Haonan Tang, Yuliang Gao, Lifeng Zhang

**Affiliations:** 1 Graduate School of Engineering, Kyushu Institute of Technology, Kitakyushu 8040015, Japan; 2 College of Animal Science and Technology, Yangzhou University, Yangzhou 225009, China; 3 College of Information and Artificial Intelligence, Yangzhou University, Yangzhou 225012, China

**Keywords:** underwater fish detection, structure-driven feature modeling, multi-scale structural learning, YOLO-based architectures

## Abstract

Accurate fish detection in complex underwater environments remains challenging due to scattering, low contrast, blur, occlusion, and background interference, which significantly degrade appearance-based representations. To address these issues, this study develops a progressive Structure-Driven Feature Modeling mechanism for underwater fish detection. The proposed mechanism extracts multi-scale smoothed residuals from shallow features, normalizes their responses, and uses the resulting structural representation to guide spatial feature modulation, while retaining complementary appearance information through feature fusion. To implement this framework, a set of Structure-Aware Feature Modeling Units is designed, integrating Spectral Encoder, Structure Channel Attention, Multi-Scale Structure Difference, Structure Normalization Module, Structure-Guided Spatial Attention, and Refine. These modules collaboratively enhance structural representations across channel and spatial dimensions. Furthermore, a Structure-Driven Feature Modeling method and a Structure-Driven Fusion strategy are introduced to facilitate progressive multi-scale structural learning based on the proposed Structure-Aware Feature Modeling Units, while enabling complementary interaction between structural and appearance features. To support evaluation, a challenging underwater fish detection dataset, Medaka, is constructed, covering occlusion, illumination degradation, and complex background interference. Extensive controlled experiments demonstrate that the proposed method can be integrated into multiple YOLO-based architectures and consistently improves detection performance under the same dataset partition and evaluation protocol. Specifically, on YOLOv8n, mAP@0.5 increases from 0.6332 to 0.6647 (+3.15 pp), and mAP@0.5:0.95 improves from 0.2495 to 0.2691 (+1.96 pp), while Precision and Recall are enhanced by 7.55 pp and 3.80 pp, respectively. Consistent gains are also observed on YOLOv5n, YOLOv7-tiny, YOLOv10n, YOLOv11n, and YOLOv12n, supporting the effectiveness and cross-architecture transferability of the proposed Structure-Driven Feature Modeling mechanism within the evaluated YOLO family. Additional evaluation on the public Blue Bot Dataset showed that Structure-Driven YOLOv8n improved mAP@0.5 and mAP@0.5:0.95 over YOLOv8n by 3.36 pp and 1.85 pp, respectively. Overall, the proposed framework provides an effective solution for structure-aware feature learning in the evaluated underwater detection settings, particularly for challenging cases involving small objects, blurred boundaries, occlusions, and visually similar targets.

## Simple Summary

Accurate fish monitoring is important for aquaculture management and animal observation, but detecting fish in underwater images is difficult when they are small, blurred, partly hidden, poorly illuminated, or visually similar to the background. This study developed an image-based detection method that places greater emphasis on fish boundaries, shapes, and body structure while retaining useful appearance information. The method was evaluated using a newly constructed dataset of Medaka, also known as Japanese rice fish, collected under challenging rearing conditions. The proposed method improved detection accuracy compared with the original detection model and produced consistent improvements when applied to several related detection models. The method was also evaluated on a public underwater fish dataset containing four different fish species, where it again improved detection performance compared with the original model. These results indicate that emphasizing structural information can help computers recognize fish more reliably when their visual appearance is unclear or partly obstructed. The proposed method may support more accurate and efficient fish monitoring, reduce the need for continuous manual observation, and provide useful technical assistance for aquaculture management and studies of fish behavior.

## 1. Introduction

Monitoring fish populations in natural aquatic environments is essential for ecosystem assessment, biodiversity conservation, and sustainable fisheries management [1]. Fish abundance, spatial distribution, and behavior serve as key indicators of ecosystem health, enabling long-term evaluation of environmental changes and human impacts [2]. In recent years, vision-based underwater monitoring has emerged as an effective complement to traditional sampling methods [3]. As a non-intrusive approach, it enables continuous, large-scale, and automated observation without disturbing natural fish behavior [4].

However, reliable fish detection in underwater environments remains challenging. Unlike terrestrial scenarios, underwater imaging can be affected by light absorption and scattering [5,6], leading to reduced local contrast and image clarity. Meanwhile, scattering and suspended particles degrade structural details and edge information, resulting in texture deterioration and blurred target boundaries [7,8]. Consequently, fish targets often exhibit low contrast, blurred boundaries, and low saliency, making them difficult to distinguish from complex backgrounds such as rocks, vegetation, and debris. These degradations are particularly severe in complex underwater environments and directly hinder reliable perception.

To address these challenges, numerous underwater fish detection methods have been proposed [9,10], most of which are adapted from general object detection frameworks designed for terrestrial scenes [11,12,13]. Among these general-purpose frameworks, Faster R-CNN is a representative two-stage detector that integrates region proposal generation with region-wise classification and bounding-box regression in a unified detection architecture [14]. These approaches typically rely on appearance cues, such as color contrast and texture patterns, with limited modifications to existing architectures [15,16]. While effective under favorable conditions, their performance degrades significantly in complex underwater environments [17]. In practice, fish are frequently occluded, captured under uneven illumination, or exhibit high similarity to surrounding objects. Small fish are also easily confused with suspended particles. Under such conditions, blurred target boundaries and target–background similarity weaken discriminative appearance cues, while texture degradation reduces feature reliability. As a result, appearance-driven methods often fail when target saliency is low or background interference is strong, indicating the limitations of directly applying conventional detection strategies.

Existing studies have attempted to improve detection performance from three main perspectives. The first introduces underwater image enhancement prior to detection to improve visual quality through color correction and contrast enhancement [18,19]. However, these methods are primarily designed for human perception and may not align with the requirements of vision models [20]. Their effectiveness is often limited and sensitive to environmental variations, leading to poor generalization across different datasets [21,22].

The second focuses on enhancing color or texture representations at the feature level using attention mechanisms [23,24,25,26]. Although these approaches improve feature discriminability to some extent, they remain dependent on appearance cues [27]. In complex underwater environments, where degradation is coupled and severe, modeling color or texture alone is insufficient to resolve ambiguity between targets and background.

The third incorporates context modeling, such as global context or long-range dependencies, to improve semantic consistency [28,29,30,31,32,33]. While these methods expand the receptive field and enhance feature integration, they may also introduce irrelevant background information [34]. In scenarios with strong interference and low target saliency, excessive reliance on contextual information can suppress target-specific features and limit detection performance.

Overall, existing approaches based on image enhancement, appearance modeling, and context modeling remain insufficient for complex underwater environments [35]. Image enhancement is sensitive to environmental variations [36], appearance-based methods are vulnerable to degradation and noise [37,38], and context modeling may introduce redundant background information [39]. These approaches largely depend on cues that are not intrinsically tied to the biological characteristics of fish, resulting in limited robustness under challenging conditions.

In contrast, structural information, such as contours and morphological characteristics, is less sensitive to illumination and color variations and thus more robust in degraded environments [40]. Compared with fine-grained color and texture cues, shape- and part-based representations can provide complementary and relatively stable information under appearance variations and partial occlusion [41,42]. Therefore, explicitly modeling fish boundaries, contours, and morphological organization may provide a more reliable discriminative basis for detection in degraded underwater scenes. Motivated by this observation, this study develops a Structure-Driven Feature Modeling mechanism that reinforces structural cues while retaining complementary appearance information through feature fusion [43,44].

Building upon the above analysis, this study proposes a structure-driven feature enhancement method to improve the robustness of fish detection in complex underwater environments. Specifically, “structure-driven” refers to explicitly constructing structural responses sensitive to fish boundaries, contours, and geometric variations, and employing them as dominant signals for feature modulation. This design shifts the model focus from appearance-dependent cues (e.g., color and texture), which are highly vulnerable to scattering and illumination variations, to structurally meaningful representations with stable geometric properties.

On this basis, a progressive modeling framework is developed from a mechanistic perspective, consisting of three stages: structure separation, structure modeling, and structure enhancement. Low-frequency suppression is first introduced to alleviate scattering-induced blur, followed by multi-scale structural modeling to compensate for occlusion-induced information loss. Finally, structure-guided spatial modulation is employed to enhance target–background discriminability. This design establishes a clear transition from conventional appearance restoration to structure-oriented feature learning.

Following this principle, a set of collaboratively designed structure-aware modules is constructed to progressively characterize and reinforce structural representations. A lightweight Spectral Encoder is first introduced to improve local continuity and distribution consistency, providing stable inputs for subsequent processing. Then, a Structure Channel Attention (SCA) module performs channel-wise recalibration, emphasizing responses sensitive to morphological variations while suppressing unstable appearance features. To explicitly disentangle structural information, a Multi-Scale Structure Difference (MSD) module extracts smoothed residuals across multiple scales, effectively separating low-frequency background components from high-frequency structural cues. To address scale-wise distribution inconsistency, a Structure Normalization Module (SNM) is further incorporated to align structural responses across spatial locations and scales, thereby improving robustness under occlusion conditions. Furthermore, a Structure-Guided Spatial Attention (SGSA) module aggregates multi-scale structural features to generate spatial attention maps, enabling feature responses to be dominated by structurally salient regions while suppressing irrelevant background interference. A lightweight refinement module with residual connections is finally employed to stabilize feature outputs, ensuring that original semantic information is preserved while enhancing structural consistency.

Based on the above structural modeling pipeline, a Structure-Driven Feature Modeling method is designed to embed structure-oriented principles into the detection process, enabling progressive transformation from raw features to structure-enhanced representations. Meanwhile, to prevent the loss of useful appearance information, a Structure-Driven Fusion strategy is introduced to jointly model original and structure-enhanced features along the channel dimension, followed by lightweight reorganization for adaptive fusion. This design achieves a balanced representation that preserves informative color and texture cues while maintaining strong structural discriminability.

The proposed method is integrated into the feature pyramid of YOLOv8, specifically at the high-resolution P3 level, to enhance small-object perception under complex backgrounds. Without relying on explicit image enhancement, the proposed framework is designed to mitigate the adverse effects of scattering, occlusion, and appearance similarity, thereby improving detection robustness under the underwater conditions represented in the evaluated datasets.

The main contributions of this study can be summarized as follows:

(1) Construction of a challenging Medaka fish detection dataset (Data Contribution). A Medaka dataset is constructed for fish detection under the imaging conditions represented in the collected larval breeding environment. The dataset contains challenging cases involving small targets, partial occlusion, uneven illumination, reduced contrast, blur, and complex background interference.
(2) Development of a progressive Structure-Driven Feature Modeling mechanism (Methodological Contribution).
  The proposed mechanism derives multi-scale structural responses directly from shallow intermediate features through smoothed residual extraction. The responses are normalized across scales and used to guide spatial feature modulation, forming a progressive feature-space modeling process for reinforcing boundary, contour, and morphological cues. To the best of our knowledge, this specific coordination of multi-scale structural difference modeling, response normalization, and structure-guided spatial modulation has not been explicitly investigated for underwater fish detection.
(3) Coordinated modeling and fusion at the high-resolution P3 level (Architectural Contribution). The Structure-Aware Feature Modeling Units are arranged in a cascaded manner at the high-resolution P3 level to progressively reinforce structural cues. A Structure-Driven Fusion strategy subsequently combines the enhanced representation with the original appearance features. The contribution lies in the task-oriented organization and interaction of these operations rather than in claiming that the individual attention, residual extraction, normalization, or fusion operations are independently novel.
(4) Controlled cross-architecture validation (Experimental Contribution).
  The proposed Structure-Driven Feature Modeling mechanism is integrated into YOLOv5n, YOLOv7-tiny, YOLOv8n, YOLOv10n, YOLOv11n, and YOLOv12n. Under a unified dataset partition, training strategy, checkpoint selection criterion, and evaluation protocol, controlled paired comparisons between each original architecture and its structure-driven counterpart consistently show performance improvements. These results support the effectiveness and cross-architecture transferability of the proposed mechanism within the evaluated YOLO family.

## 2. Materials and Methods

### 2.1. Medaka Dataset Construction

In the controlled but visually challenging Medaka tank environment considered in this study, fish targets exhibit substantial variability in visibility, scale, occlusion, and background interference. The dataset was collected from a Medaka (Oryzias latipes) larval-breeding tank at the Kyushu Institute of Technology to represent challenging underwater imaging conditions involving aquatic vegetation, suspended particles, uneven illumination, reduced contrast, and blur. Within this environment, fish targets are frequently subject to partial occlusion by aquatic vegetation, duckweed, and suspended debris. Meanwhile, due to light absorption and scattering in water, the captured images commonly exhibit uneven illumination, reduced contrast, and varying degrees of blur.

Furthermore, Medaka fish are inherently small in size and exhibit a certain degree of transparency, making them highly confusable with background elements such as aquatic vegetation, sediment, rocks, and suspended particles. Small-scale targets are particularly prone to being misidentified as floating debris, which further increases detection difficulty. As a result, the dataset is characterized by strong background interference, a high proportion of small and low-contrast targets, and evident imaging degradation, thereby imposing more stringent requirements on the robustness and structural representation capability of detection models.

**Figure 1:**
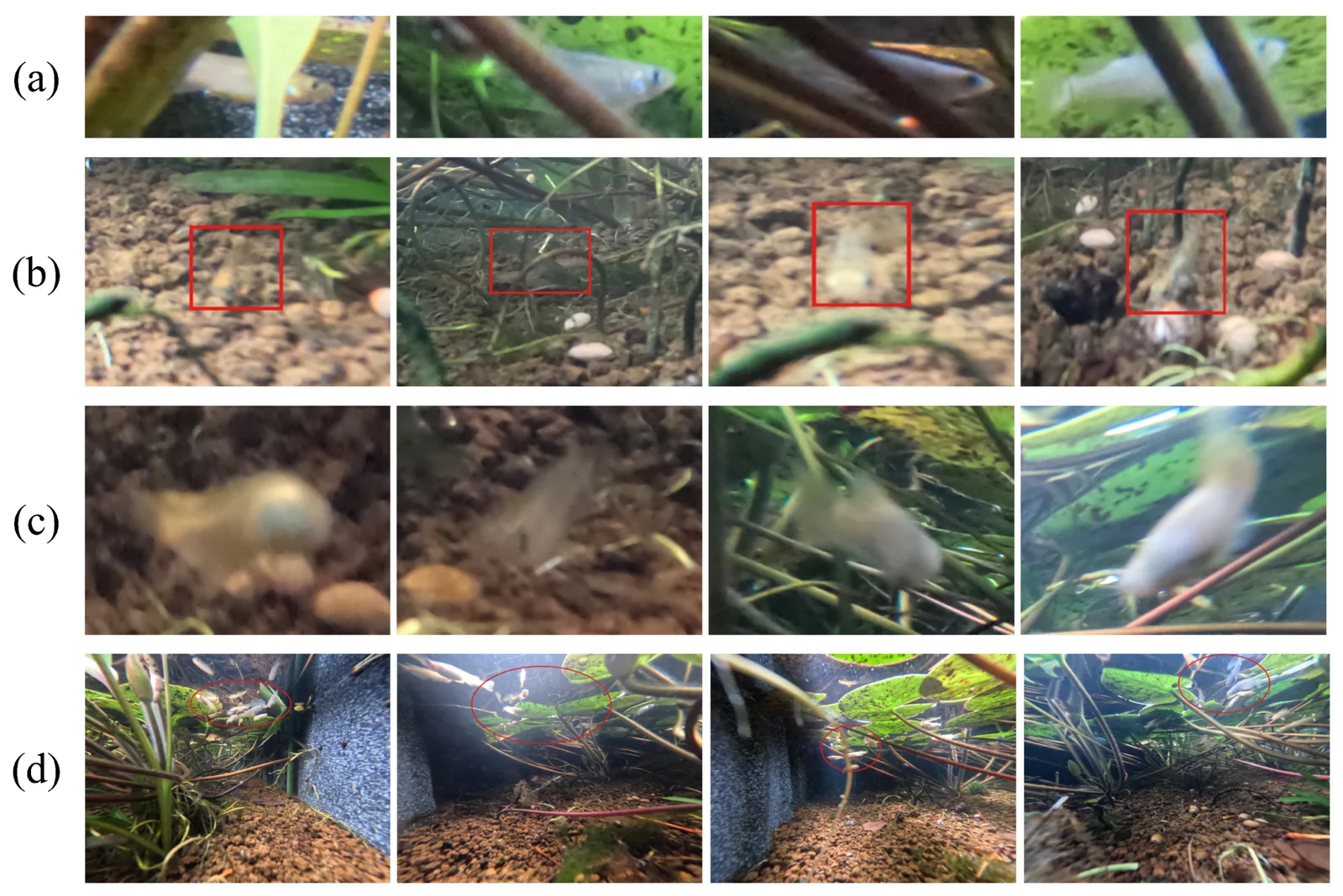
Challenging samples include: (**a**) occlusion, (**b**) similarity, (**c**) blur, (**d**) local crowding.

As shown in Figure 1, several representative challenging samples are presented, including cases of severe occlusion, high visual similarity, target blur, and local crowding. These examples further demonstrate the difficulty and practical relevance of the constructed dataset. Overall, the dataset provides a challenging benchmark for evaluating structure-aware fish detection methods under the imaging conditions represented in the collected Medaka tank environment.

#### 2.1.1. Data Collection

The dataset was collected using a GoPro HERO11 Black (GoPro, San Mateo, CA, USA) in underwater environments. All images were captured at a spatial resolution of 1920 × 1080 pixels. A time-lapse acquisition strategy was adopted, capturing one frame every 20 s to ensure temporal continuity and dynamic diversity. This interval describes the original image acquisition process; temporally adjacent frames were not directly retained as samples in the final dataset. To further enhance spatial diversity, the camera position and shooting angle were adjusted every two hours, enabling the capture of fish distributions under varying perspectives and background conditions. For each fixed camera position and viewing angle, only a small number of representative images captured at separated time points and under different illumination conditions were retained.

Along the temporal dimension, data collection spanned multiple periods from 08:00 to 18:00, incorporating variations in illumination intensity and incident angles to better reflect realistic underwater imaging conditions. The experimental tank contained artificially planted aquatic vegetation and duckweed, with a sand substrate at the bottom, along with a certain amount of suspended particles and impurities. These factors jointly constituted a complex and representative underwater environment.

**Figure 2:**
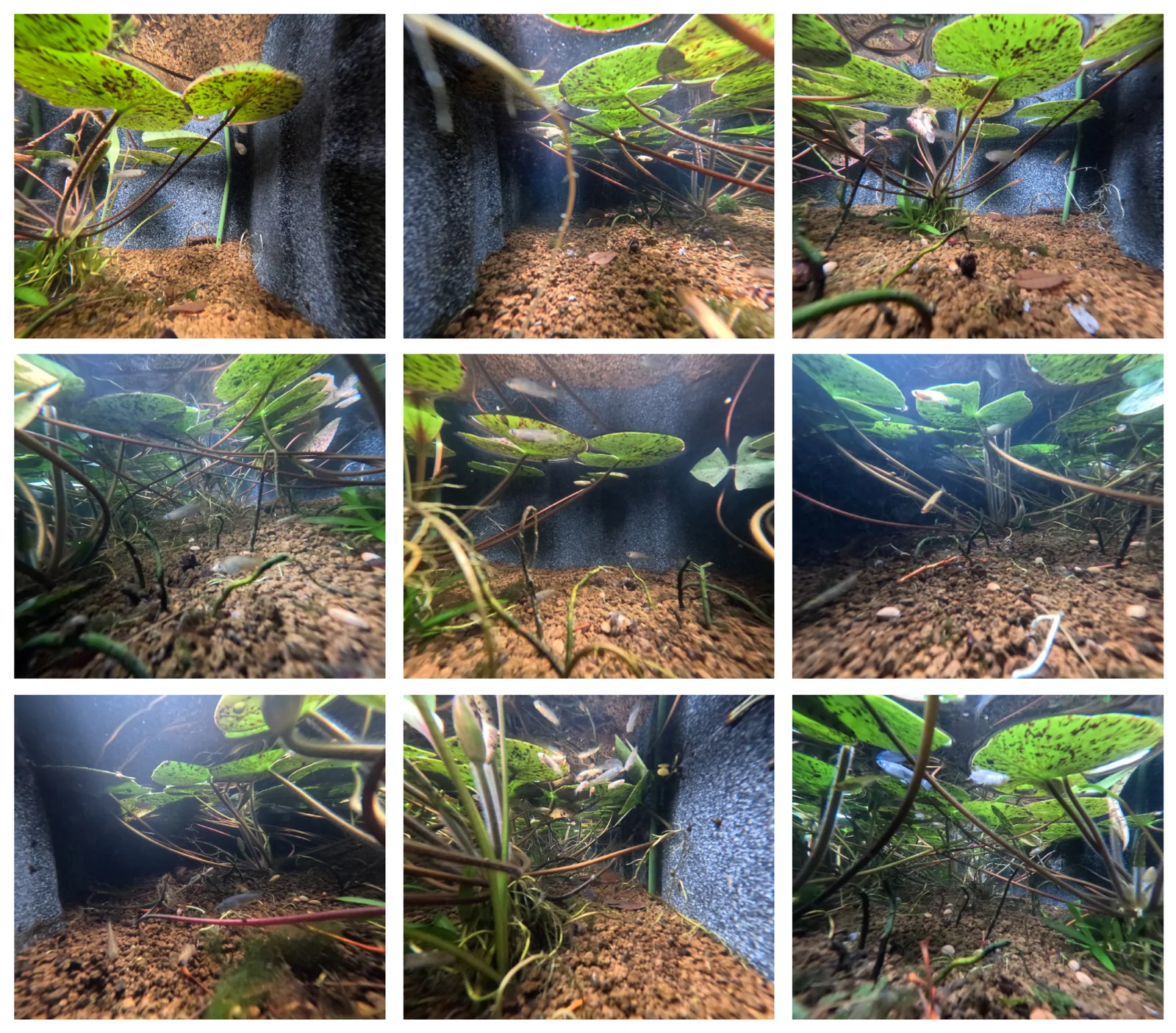
Representative samples captured under different imaging positions, viewing angles, and illumination conditions across different time periods.

As illustrated in Figure 2, representative samples captured under different imaging positions, viewing angles, and illumination conditions at different times are presented. Fish targets in the collected images are typically distributed among the stems and leaves of aquatic plants, with some instances subject to partial occlusion. The background also contains fine-grained textures and suspended particles. In addition, due to refraction and scattering effects in water, certain images exhibit slight blur and uneven illumination. These factors reduce the discriminability between targets and background, thereby significantly increasing the difficulty of the detection task.

#### 2.1.2. Data Processing and Annotation

During the data preprocessing stage, the raw images were first filtered and curated to remove severely blurred samples or those without valid targets, ensuring overall data quality. Temporally adjacent and visually redundant frames captured from the same camera position were also excluded, and only representative images acquired at separated time points and under different illumination conditions were retained. The images were then resized to a uniform resolution to meet the input requirements of model training. After this curation process, the final dataset consists of 2080 images, each containing between 2 and 8 fish targets, exhibiting evident multi-object and scale variation characteristics. The curated dataset was subsequently randomly divided into training, validation, and test sets with a ratio of 7:2:1. Accordingly, the training subset contained 1456 images and several thousand annotated fish instances, providing denser object-level supervision than the image count alone may suggest. In addition, to reduce overfitting and improve robustness to appearance and scale variations within the training distribution, common data augmentation strategies—such as random flipping, scale transformation, and color perturbation—were employed during training.

During the annotation stage, fish targets were manually labeled on an instance-by-instance basis. Bounding boxes were annotated using the LabelImg tool, and the annotations were converted from Pascal VOC format to YOLO-format labels. To emphasize the importance of structural information, unified annotation criteria were adopted for various complex cases. Specifically, fish appearing in water-surface reflections were included, as their overall structural contours remain discernible despite blurred texture details. Similarly, fish with blur or partial occlusion were annotated as long as their essential structural characteristics remained discernible. In contrast, targets with more than two-thirds of their bodies occluded were excluded to reduce the influence of highly uncertain samples on model training.

### 2.2. Baseline Architecture

In this study, YOLOv8 [45] is adopted as the baseline detection architecture. YOLOv8 follows a one-stage, anchor-free design, consisting of a backbone network, a feature aggregation neck, and a fully decoupled detection head. During prediction, the model directly performs object center localization and bounding box regression, while separating classification and localization tasks to improve optimization stability and reduce task interference.

Compared with YOLOv5 [46], YOLOv8 replaces the conventional C3 module in the backbone with the C2f module. The C2f module enhances feature reuse and internal information flow through gradient flow partitioning, while maintaining a controllable parameter scale. In the feature fusion stage, YOLOv8 simplifies the PAN-FPN structure by reducing redundant convolutional operations, thereby improving multi-scale feature propagation efficiency. In contrast to YOLOv7 [47], which adopts the E-ELAN structure to expand gradient paths and relies on anchor-based prediction, YOLOv8 employs a more modular design and adopts anchor-free regression, reducing dependence on anchor-related hyperparameters and simplifying the sample assignment process.

Compared with more recent variants such as YOLOv10 [48], YOLOv11 [49], and YOLOv12 [50], although these models introduce further optimizations in training strategies and inference efficiency, YOLOv8 maintains a favorable balance in terms of architectural maturity, implementation stability, and module extensibility. In complex underwater scenarios, detection performance depends not only on model capacity and computational efficiency, but also critically on the stability of feature representation. Owing to its clear modular structure and stable optimization behavior, YOLOv8 provides a reliable and extensible foundation for structure-aware feature modeling and enhancement at the feature level.

### 2.3. Overview of the Proposed Structure-Driven YOLOv8 Architecture

In the standard YOLOv8 architecture, the backbone outputs three feature maps at different scales, denoted as F3, F4, and F5, corresponding to hierarchical representations with different downsampling rates. Among them, F3 has the highest spatial resolution and preserves richer local details, while F5 exhibits the highest level of semantic abstraction. These features are then fed into the neck for top-down and bottom-up multi-scale fusion, generating prediction features P3, P4, and P5, which are subsequently passed to the detection head for multi-scale object prediction. Notably, P3, with the highest resolution, is primarily responsible for small-object detection and is particularly sensitive to spatial details and local structural variations.

Although this multi-scale design effectively integrates semantic and detailed information, its feature modeling process largely relies on the statistical distribution of convolutional responses, lacking explicit modeling of spatial structural organization. This limitation is particularly evident in the P3 layer, where feature responses are predominantly governed by color and texture cues, while structural patterns are insufficiently emphasized.

**Figure 3:**
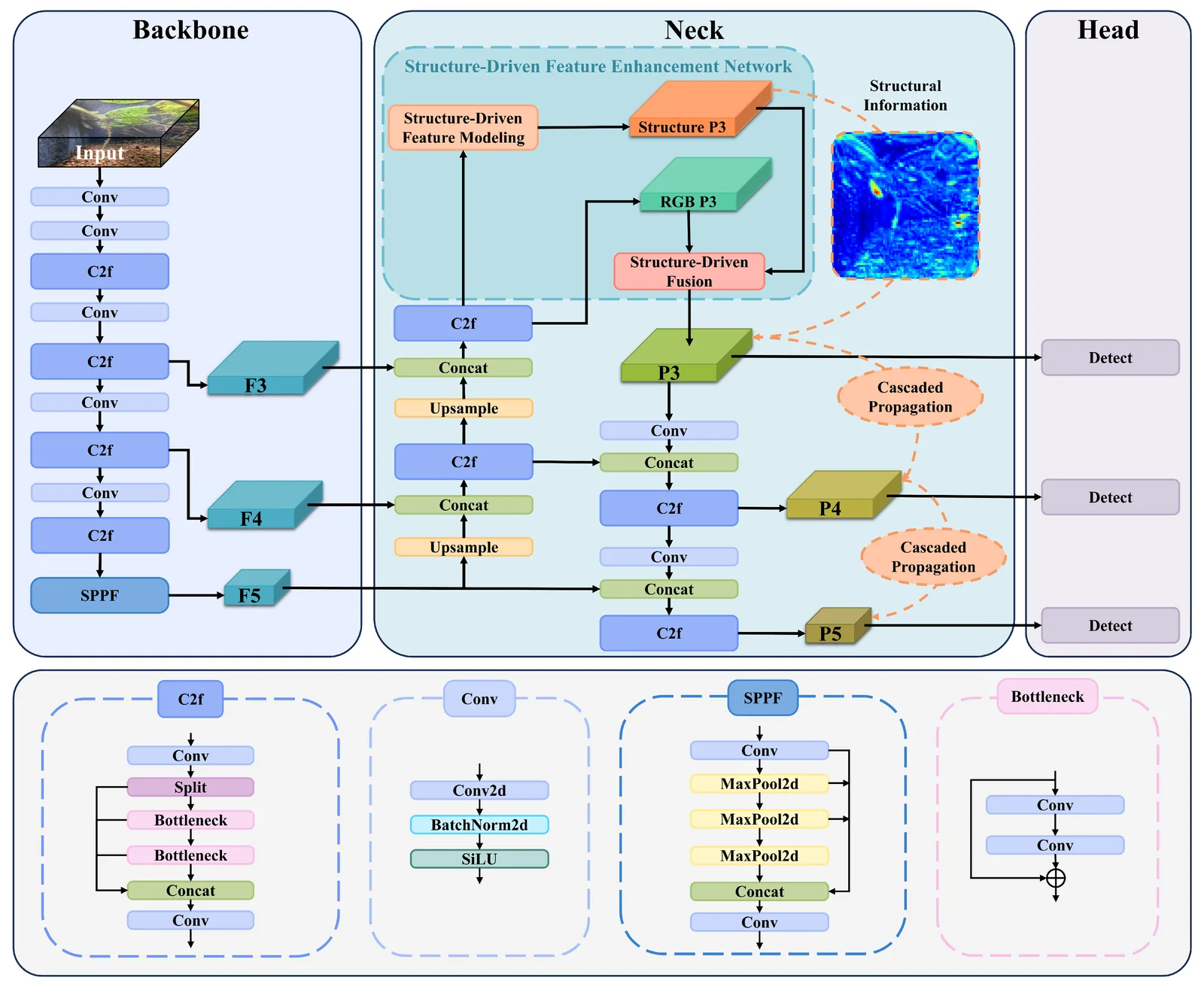
Overview of the proposed Structure-Driven YOLOv8 architecture.

To address this issue, a Structure-Driven Architecture is proposed based on YOLOv8. Without altering the backbone or the overall multi-scale prediction pipeline, a Structure-Driven Feature Enhancement Network is introduced during the generation of P3 from F3, enabling structure-aware reconstruction and guided modeling of shallow high-resolution features, as illustrated in Figure 3.

Specifically, F3 is first processed by the Structure-Driven Feature Modeling component to extract structural patterns, producing a structure-enhanced feature map denoted as Structure P3. Meanwhile, the original neck generates the conventional P3, which is dominated by RGB appearance features and is denoted as RGB P3. These two representations are then fused in the Structure-Driven Fusion component to produce the final enhanced P3.

This fusion process strengthens spatial contours and morphological organization within P3, and adaptively reweights channel-wise and spatial responses. As a result, the enhanced P3 not only preserves multi-scale semantic representation capability but also increases the contribution of structural information in shallow features, while suppressing non-structural responses dominated by color and texture. Consequently, a more stable and discriminative structural representation is obtained. The enhanced P3, together with P4 and P5, participates in the final multi-scale prediction, forming a structure-enhanced detection pipeline.

### 2.4. Structure-Driven Modeling Principle

In complex underwater environments, the discriminability of fish targets is jointly affected by multiple imaging degradation factors. Fish are often partially occluded by aquatic vegetation, duckweed, and suspended particles, while light absorption and scattering in water introduce significant image degradation, including uneven illumination, reduced contrast, and varying degrees of blur. In addition, fish frequently exhibit high similarity to background elements—such as rocks, aquatic plants, and sandy substrates—in terms of color and texture, and small-scale targets are easily confused with suspended particles. These factors collectively result in strong background interference, weakened structural cues, and noticeable degradation in image quality, making it difficult for appearance-based feature representations to reliably distinguish targets from the background.

From the perspective of feature representation, the essence of the above problem lies in the dominance of low-frequency components—such as illumination, color, and blur—induced by underwater scattering, while discriminative high-frequency structural information, including boundaries, contours, and morphological variations, is significantly weakened or even suppressed. However, standard convolution primarily relies on local weighted aggregation for response statistics, tending to emphasize intensity information, and lacks the capability to explicitly distinguish structural variations from smooth background regions. Therefore, in complex underwater scenarios, effectively separating and enhancing structural information from mixed feature representations becomes a critical challenge for improving detection robustness.

To address this issue, a structure-driven modeling mechanism is proposed, which constructs a progressive process in the feature space consisting of “structure separation–structure modeling–structure enhancement”, enabling the gradual extraction and reinforcement of structural information.

First, in the structure separation stage, local spatial smoothing is employed to explicitly model low-frequency background components induced by underwater scattering, and structural responses are extracted in a residual form. This process suppresses blur and low-contrast interference, shifting feature representation from low-frequency dominance to structure dominance.

Second, in the structure modeling stage, multi-scale structural modeling and normalization mechanisms are introduced to extract and align structural responses across different spatial scales. Cross-scale fusion is further applied to compensate for incomplete structures caused by local occlusions, thereby improving the continuity and robustness of structural representations.

Finally, in the structure enhancement stage, the aggregated structural representation is used to generate spatial attention weights, enabling adaptive enhancement of structurally salient regions while suppressing responses in non-structural areas. This shifts the feature representation from reliance on appearance cues such as color and texture to structural differences, effectively addressing the high similarity between targets and background.

Specifically, let the input feature map be denoted as $F\in R^{C\times H\times W}$. To characterize the low-frequency smooth background induced by underwater scattering, a spatial smoothing operator with kernel size *k* is introduced:

(1) $$A_{k}\left(F\right)={\mathrm{AvgPool}}_{k\times k}\left(F\right)$$

This operator approximates low-frequency components through local mean aggregation. Based on this, the structural residual is defined as:

(2) $$D_{k}\left(F\right)=\left|F-A_{k}\left(F\right)\right|,\;k\in \left\{3,5,7\right\}$$

By subtracting the locally smoothed response, low-frequency components in the feature map are effectively suppressed, while regions with spatial variations are highlighted, thereby achieving an initial separation of structural information.

Considering that a single scale is insufficient to fully describe target structures—especially under partial occlusion caused by aquatic vegetation and suspended particles, where local structures are often incomplete or discontinuous—a multi-scale structural modeling mechanism is introduced. Structural responses are modeled over a set of scales $\left\{k_{1},k_{2},\ldots ,k_{K}\right\}$, and spatial mean normalization is applied to eliminate magnitude discrepancies across different scales:

(3) $$\hat{D_{k}}\left(F\right)=\frac{D_{k}\left(F\right)}{\frac{1}{HW}\sum_{i,j}D_{k}\left(F\right)\left(i,j\right)+\varepsilon }$$

where ε is a numerical stability term. Based on this, a unified structural representation is obtained via cross-scale fusion:

(4) $$S\left(F\right)=\frac{1}{K}\sum_{k}\hat{D_{k}}\left(F\right)$$

This process integrates local and global structural information under a larger receptive field, enabling structural cues captured at different scales to complement each other, thereby forming more continuous and complete structural representations under occlusion conditions.

After obtaining a stable structural representation, a structure-guided spatial attention mechanism is further constructed. The structural response is fused with feature representations and mapped into spatial attention weights:

(5) $$M\left(F\right)=\sigma \left(\Phi \left(\mathrm{Concat}(F_{c},S\left(F\right))\right)\right)$$

where Φ(·) denotes a transformation that compresses multi-channel features into a single-channel spatial response map, and σ(·) represents the Sigmoid activation function. The corresponding dimensional relationships are $F_{c}\in R^{C\times H\times W}$, $S\left(F\right)\in R^{C\times H\times W}$, and $M\left(F\right)\;\in \;R^{1\times H\times W}$.

Based on this, residual-based modulation and refinement are applied to the features, yielding the final enhanced representation:

(6) $$F^{\prime }=F+\mathrm{Refine}\left(F_{c}⊙\left(1+\beta M(F)\right)\right)$$

where ⊙ denotes element-wise multiplication, and β is a learnable scaling coefficient.

From a signal processing perspective, the above process can be interpreted as a spatial-domain, frequency-inspired decomposition and information decoupling mechanism. The input feature can be expressed as the superposition of low-frequency and high-frequency components—i.e., $F=F_{\mathrm{low}}+F_{\mathrm{high}}$—where the low-frequency component corresponds to illumination, blur, and background interference, while the high-frequency component represents structural information. The spatial smoothing operator approximates the low-frequency component—i.e., $A_{k}\left(F\right)\approx F_{\mathrm{low}}$—whereas the structural residual $D_{k}\left(F\right)$ provides an approximation of the high-frequency structural information, thereby achieving effective separation between low-frequency degradation and discriminative structural cues.

From the perspective of classical vision modeling, this process is consistent with Retinex theory [51] and the Laplacian operator [52]. The former achieves illumination-invariant modeling by separating reflectance and illumination components, while the proposed method separates low-frequency and structural information through smoothing and residual operations. The latter emphasizes edge structures via local differences, whereas the proposed approach provides a more stable approximation of structural responses. Therefore, the proposed method can be regarded as a concise and differentiable implementation of classical visual decomposition principles within a deep learning framework.

In summary, the proposed structure-driven modeling mechanism progressively addresses low-frequency degradation, structural loss due to occlusion, and appearance similarity interference through a unified process of structure separation, structure completion, and structure enhancement. Specifically, it adapts low–high frequency decomposition to shallow feature modeling and coordinates it with multi-scale residual extraction, response normalization, and structure-guided spatial modulation, thereby reinforcing structural representation under the evaluated underwater conditions.

### 2.5. Construction of Structure-Driven Feature Enhancement Network

Convolutional neural networks progressively extract semantic features during forward propagation. However, in complex underwater scenes, boundary and contour cues may be weakened by blur, low contrast, local occlusion, and target–background similarity. To address this problem, we develop a Structure-Driven Feature Enhancement Network that explicitly extracts and progressively reinforces these cues.

**Figure 4:**
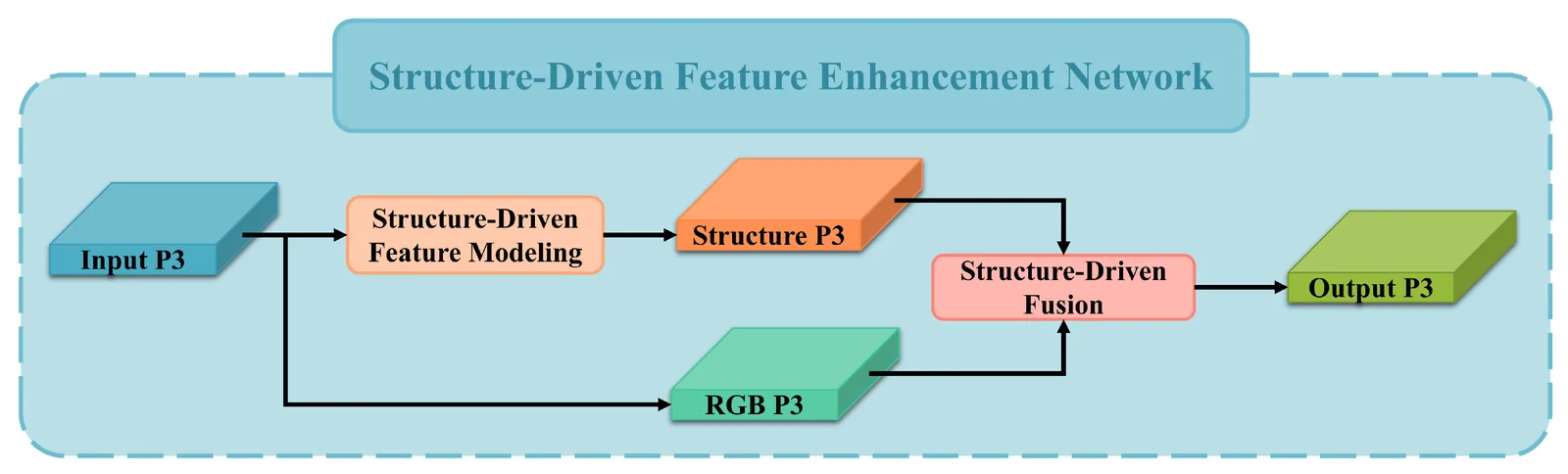
Overall framework of the proposed Structure-Driven Feature Enhancement Network.

The network consists of two collaborative components: Structure-Driven Feature Modeling and Structure-Driven Fusion. The Structure-Driven Feature Modeling component performs progressive structural enhancement following a “structure separation–structure modeling–structure enhancement” pipeline, aiming to mitigate the effects of scattering and blur, compensate for structural loss caused by occlusion, and improve structural discriminability. The Structure-Driven Fusion component, on the other hand, integrates structure-enhanced features with original appearance information under a structure-dominant paradigm, resulting in more complete and robust feature representations. The overall architecture is illustrated in Figure 4.

#### 2.5.1. Design of Structure-Aware Feature Modeling Units and Construction of Structure-Driven Feature Modeling Component

**Figure 5:**
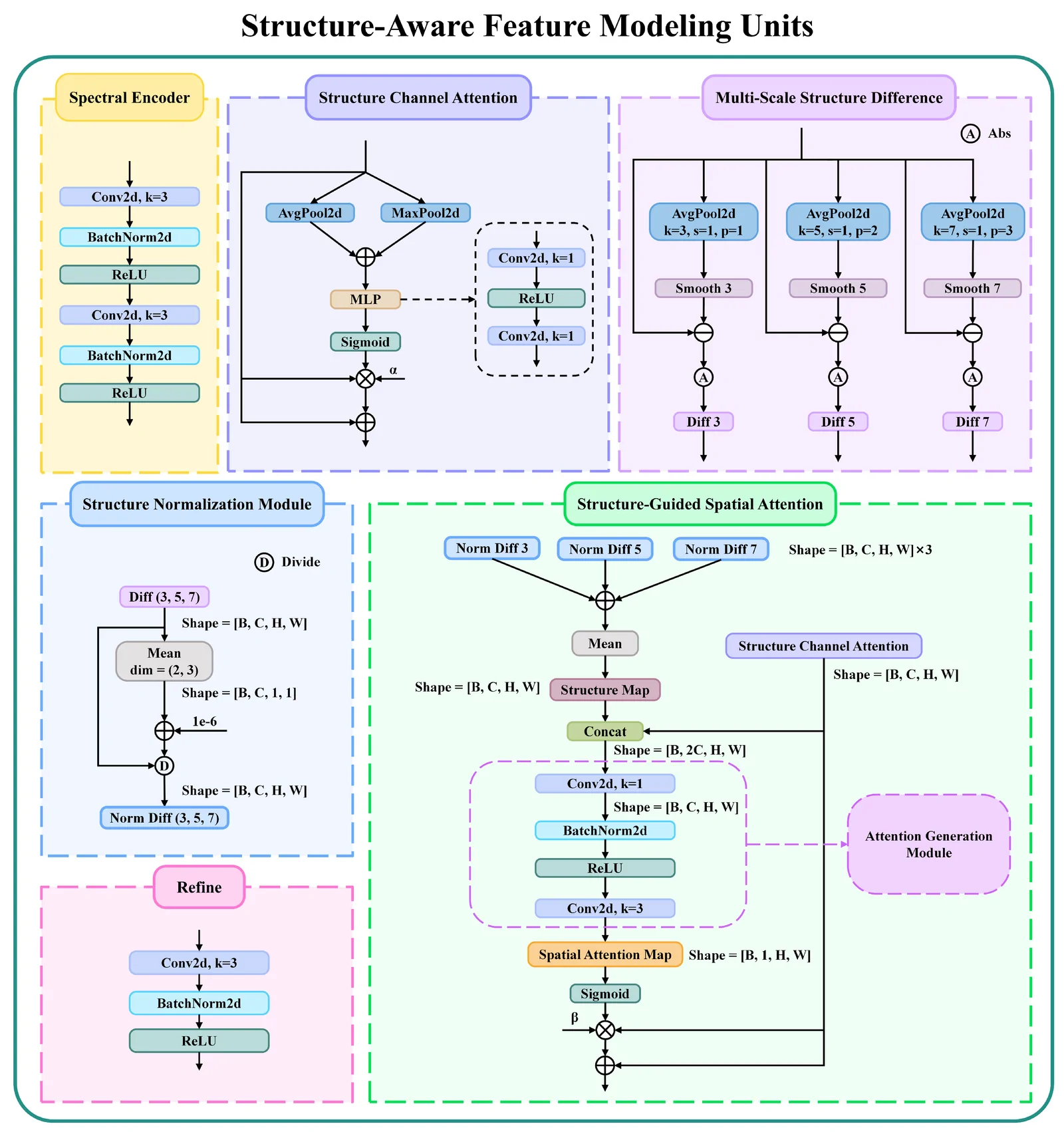
Structural design of the proposed Structure-Aware Feature Modeling Units.

**Figure 6:**
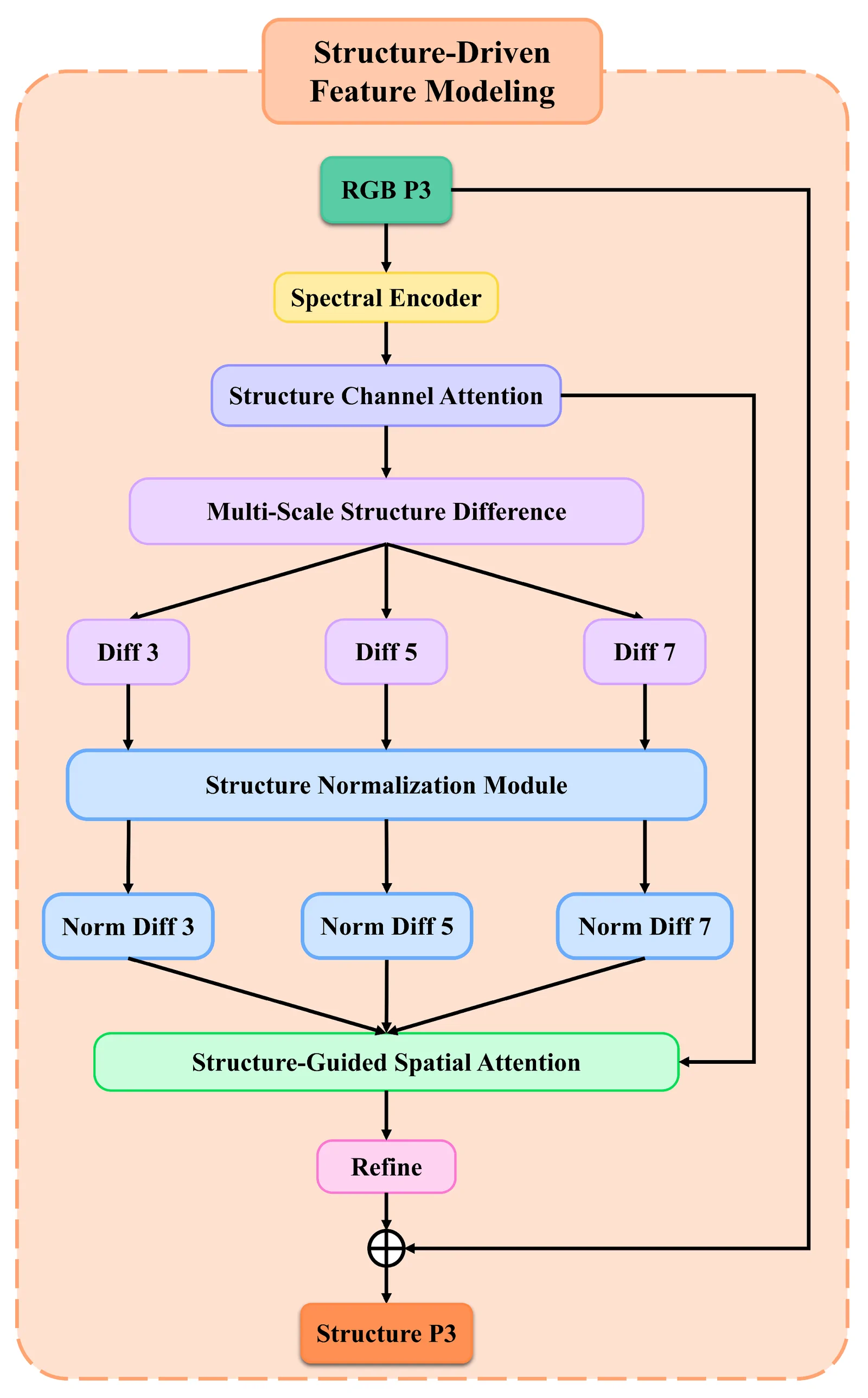
Architecture of the proposed Structure-Driven Feature Modeling component.

The Structure-Driven Feature Modeling component progressively processes the P3 feature extracted from the backbone. It consists of lightweight Structure-Aware Feature Modeling Units that perform multi-scale residual extraction, response normalization, and spatial attention, as illustrated in Figure 5. These units are arranged in a cascaded manner to reinforce boundary, contour, and morphological cues under complex underwater degradation conditions, as illustrated in Figure 6.

During the structure separation stage, the input features are first shallowly re-encoded through a Spectral Encoder module. Specifically, two consecutive 3×3 convolutional layers, followed by Batch Normalization and ReLU activation, are employed to enhance local continuity and distribution consistency, thereby suppressing noise interference in the original features and producing stable re-encoded features $F_{s}$.

On this basis, a Structure Channel Attention module is introduced to adaptively recalibrate channel responses and emphasize structure-sensitive feature representations. Specifically, global average pooling and global max pooling are jointly utilized to model channel-wise statistical information, and channel weights are generated through a lightweight mapping function:

(7) $$a=\sigma \left({\mathrm{Conv}}_{2}\delta \left({\mathrm{Conv}}_{1}\left(\mathrm{GAP}\left(F_{s}\right)+\mathrm{GMP}\left(F_{s}\right)\right)\right)\right)$$

Subsequently, residual-style channel modulation is applied to enhance structure-related channels:

(8) $$F_{c}=F_{s}⊙\left(1+\alpha a\right)$$

This design enhances the representation capability of structure-sensitive channels while preserving the stability of the original feature distribution, thereby providing clearer structural responses $F_{c}$ for subsequent multi-scale structural modeling.

Subsequently, Multi-Scale Structure Difference module is employed to construct smoothed residuals across multiple scales, explicitly separating low-frequency background components from feature representations. This process enables the extraction of stable structural responses under varying degrees of blur, preserving and strengthening discriminative information such as boundaries and contours, thereby effectively alleviating the influence of underwater scattering and blur degradation. The core operation constructs multi-scale structural differences as follows:

(9) $$D_{k}=\left|F_{c}-{\mathrm{AvgPool}}_{k\times k}\left(F_{c}\right)\right|,\;k\in \left\{3,5,7\right\}$$

During the structure modeling stage, to alleviate local occlusion caused by underwater plants and suspended particles, a Structure Normalization Module is introduced to normalize the multi-scale structural differences—namely, Diff 3, Diff 5, and Diff 7—thereby improving the comparability of structural responses across different scales. This process aligns structural responses with varying visibility levels, enabling collaborative and balanced fusion of multi-scale structural information while preventing strong responses from visible regions from suppressing weak structural responses. The core operation is the mean-normalized multi-scale structural difference, defined as follows:

(10) $$\hat{D_{k}}=\frac{D_{k}}{\frac{1}{HW}\sum_{i,j}D_{k}\left(i,j\right)+\varepsilon },\;k\in \left\{3,5,7\right\}.$$

Based on the normalized multi-scale structural differences—namely, Norm Diff 3, Norm Diff 5, and Norm Diff 7—scale aggregation is further performed to obtain the Structure Map representation:

(11) $$S\left(F_{c}\right)=\frac{1}{K}\sum_{k}\hat{D_{k}}\left(F_{c}\right)$$

By leveraging the complementarity among different scales, the proposed method enables the model to form more continuous structural representations under occlusion conditions, thereby improving the completeness and robustness of structural feature representation.

During the structure enhancement stage, to transform explicit structural responses into direct modulation of spatial feature distributions, a Structure-Guided Spatial Attention module is introduced. Specifically, structure-guided spatial attention weights are generated based on the structural representation $S(F_{c})$ and feature representation $F_{c}$:

(12) $$M\left(F_{c}\right)=\sigma \left(\Phi \left(\mathrm{Concat}\left(F_{c},S\left(F_{c}\right)\right)\right)\right)$$

Subsequently, residual-style spatial modulation is applied to enhance the feature representation:

(13) $$F_{sa}=F_{c}⊙\left(1+\beta M(F_{c})\right)$$

This mechanism employs structural differences as discriminative cues, shifting the model from appearance-based reasoning relying on color and texture toward structure-oriented discrimination based on boundaries and morphological information. Under conditions where targets and background exhibit high appearance similarity, the proposed module enhances structurally salient regions while suppressing non-structural responses, thereby reducing false detections and confusion caused by background similarity.

Finally, to stabilize the output representation, a Refine module is introduced to locally refine the attention-modulated features:

(14) $$\mathrm{Refine}\left(F_{sa}\right)=\mathrm{ReLU}\left(\mathrm{BN}\left({\mathrm{Conv}}_{3\times 3}\left(F_{sa}\right)\right)\right)$$

Since spatial attention may introduce local discontinuities or noise amplification through non-uniform weighting, the Refine module smooths and reorganizes features via neighborhood information re-aggregation, thereby improving the stability of feature representations. Subsequently, the refined features are fused with the input features through a residual connection. This operation preserves the original semantic information while injecting the enhanced representation, resulting in the final Structure P3 feature:

(15) $$F^{\prime }=F+\mathrm{Refine}\left(F_{sa}\right)$$

In summary, the Structure-Driven Feature Modeling component coordinates multi-scale smoothed residual extraction, response normalization, and structure-guided spatial modulation within a progressive feature modeling pipeline. These operations build on established concepts in residual-based structural analysis, normalization, and attention modeling. The contribution of the proposed design lies in arranging them as cascaded Structure-Aware Feature Modeling Units at the high-resolution P3 level to reinforce boundary, contour, and morphological cues under the underwater conditions evaluated in this study.

#### 2.5.2. Construction of Structure-Driven Fusion Component

**Figure 7:**
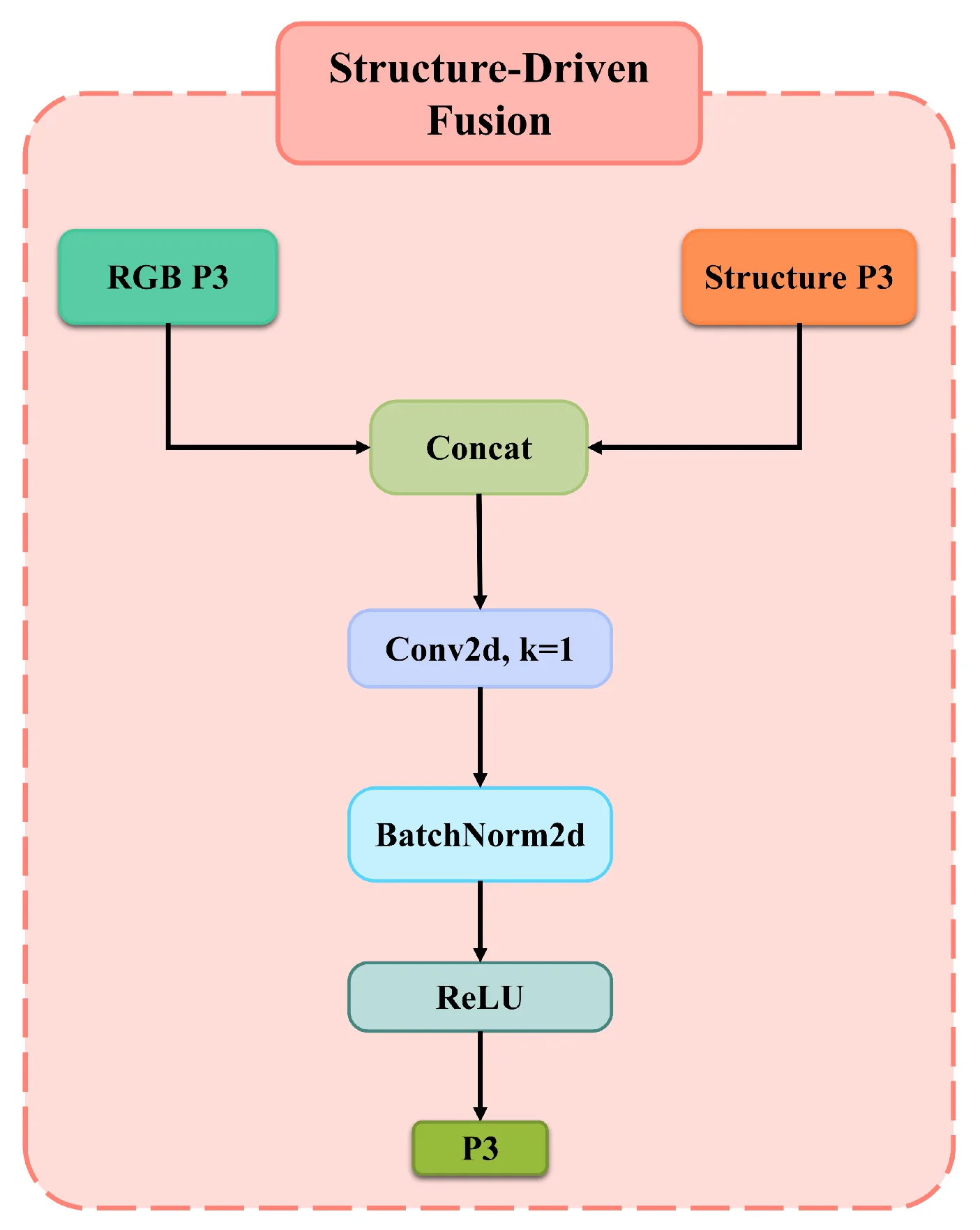
Architecture of the proposed Structure-Driven Fusion component.

Although the Structure-Driven Feature Modeling component is capable of generating structure-enhanced features, appearance information, such as color and texture, still provides complementary discriminative cues in complex underwater environments. To this end, a Structure-Driven Fusion component is designed to achieve collaborative integration of structural and appearance information under a structure-dominant scheme. Specifically, the original RGB P3 features and the Structure P3 features enhanced by the Structure-Driven Feature Modeling component are concatenated along the channel dimension. Subsequently, a 1×1 convolution followed by Batch Normalization and ReLU activation is employed for channel reorganization and nonlinear mapping, thereby enabling cross-domain feature interaction and adaptive feature fusion, as illustrated in Figure 7.

From the perspective of the modeling mechanism, structure-enhanced features provide a more robust discriminative basis against blur, low contrast, and local occlusion, whereas the original appearance features complement color, texture, and local detail information. Their integration enables the model to suppress background similarity interference through structural cues while avoiding the loss of appearance details caused by relying solely on structural modeling. Specifically, structural features effectively alleviate blur and low-contrast degradation induced by underwater scattering and provide more stable contour cues under occlusion conditions. In contrast, appearance information supplements regions where structural responses are weak or local details are insufficient, thereby maintaining the completeness of feature representations. Furthermore, when targets and background exhibit high appearance similarity, structural information remains the primary discriminative cue, while appearance information serves as complementary guidance for finer distinction.

Ultimately, the fused features preserve the original visual information while explicitly incorporating structure-enhanced responses, thereby achieving a feature representation dominated by structural cues and complemented by appearance information. This design not only maintains the model’s ability to exploit appearance information such as color and texture, but also strengthens its sensitivity to structural characteristics, including boundaries, contours, and morphological variations. Consequently, the proposed method produces more stable and discriminative detection features in complex underwater environments.

### 2.6. Evaluation Metrics

To quantitatively evaluate the performance of the proposed detection framework, standard metrics in object detection are adopted, including Intersection over Union (IoU), Average Precision (AP), Average Recall (AR), Mean Average Precision (mAP), and Mean Average Recall (mAR).

IoU measures the overlap between the predicted bounding box and the ground-truth bounding box, defined as the ratio between the intersection area and the union area:

(16) $$\mathrm{IoU}=\frac{\mathrm{Area}\left(B_{p}\cap B_{g}\right)}{\mathrm{Area}\left(B_{p}\cup B_{g}\right)}$$

where $B_{p}$ denotes the predicted bounding box and $B_{g}$ denotes the ground-truth bounding box. A detection is considered a True Positive (TP) if the IoU between the predicted and ground-truth boxes exceeds a predefined threshold; otherwise, it is regarded as a False Positive (FP). Based on detection outcomes, Precision and Recall are defined as:

(17) $$\mathrm{Precision}=\frac{\mathrm{TP}}{\mathrm{TP}+\mathrm{FP}}$$

(18) $$\mathrm{Recall}=\frac{\mathrm{TP}}{\mathrm{TP}+\mathrm{FN}}$$

where TP, FP, and FN denote the numbers of True Positives, False Positives, and False Negatives, respectively. By varying the confidence threshold of detection results, different Precision–Recall (P–R) pairs can be obtained, forming the P–R curve. The Average Precision (AP) for each class is defined as the area under the corresponding P–R curve:

(19) $$\mathrm{AP}=\int_{0}^{1}P\left(R\right)\;dR$$

To comprehensively evaluate multi-class detection performance, mean Average Precision (mAP) is adopted, which is defined as the average of AP over all categories:

(20) $$\mathrm{mAP}=\frac{1}{N}\sum_{i=1}^{N}{\mathrm{AP}}_{i}$$

where *N* denotes the number of categories and $AP_{i}$ represents the Average Precision of the *i*-th category. In this study, two commonly used mAP metrics are employed: mAP@0.5 and mAP@0.5:0.95. Specifically, mAP@0.5 is computed at a fixed IoU threshold of 0.5, while mAP@0.5:0.95 is obtained by averaging AP over multiple IoU thresholds ranging from 0.5 to 0.95 with a step size of 0.05. Compared with single-threshold metrics, mAP@0.5:0.95 provides a more comprehensive evaluation of detection performance in terms of both classification and localization accuracy. In addition, Mean Average Recall (mAR) is adopted as a complementary metric to evaluate the overall ability of the model to detect ground-truth objects. At a given IoU threshold *t*, the average recall across all categories is defined as:

(21) $${\mathrm{AR}}_{t}=\frac{1}{N}\sum_{i=1}^{N}{\mathrm{Recall}}_{i}$$

The mean Average Recall over multiple IoU thresholds is further defined as:

(22) $$\mathrm{mAR}=\frac{1}{T}\sum_{t\in T}{\mathrm{AR}}_{t}$$

where *T* denotes the set of IoU thresholds used for evaluation. The mAR metric reflects the model’s capability to detect objects under varying localization requirements from the perspective of recall.

### 2.7. Experimental Settings

All experiments were conducted on a workstation equipped with an NVIDIA GeForce RTX 3070 GPU. The training environment was built with Python 3.10.0 and PyTorch 2.1.2, with CUDA 12.1 used for hardware acceleration.

During training, the AdamW optimizer was adopted with an initial learning rate of $2\times 10^{-3}$, a momentum of 0.9, and a weight decay of $5\times 10^{-4}$. To improve training stability, a warm-up stage of 3 epochs was employed at the beginning of training, with the initial momentum set to 0.8. The batch size was set to 16, and the model was trained for a total of 300 epochs.

To ensure a unified and controlled initialization protocol, all comparative and ablation experiments were conducted without pretrained weights. This choice was made to evaluate the contribution of the proposed Structure-Driven Feature Modeling mechanism under the same randomly initialized training setting. Although unchanged layers with compatible tensor dimensions in the modified architectures can partially inherit pretrained parameters, the newly introduced structure-driven components have no corresponding pretrained weights and must be randomly initialized. Therefore, the reported results should be interpreted as controlled from-scratch comparisons rather than as the maximum performance achievable using standard pretrained initialization. The input image resolution was uniformly set to 640×640 to balance detection accuracy and computational efficiency.

For the proposed structure-aware modules, the learnable scaling coefficients in Structure Channel Attention and Structure-Guided Spatial Attention were initialized to α=0.5 and β=0.7, respectively. In addition, Mosaic data augmentation was disabled during the final 10 epochs of training to improve training stability and enhance localization accuracy.

The dataset was divided into independent training, validation, and test subsets. Model training was performed using the training set, while checkpoint selection was conducted exclusively on the validation set. For each model or experimental configuration, the checkpoint corresponding to the highest validation mAP@0.5 was selected as the final checkpoint. This checkpoint selection criterion was predefined and applied consistently throughout all comparative and ablation experiments. The selected checkpoint was subsequently evaluated on the held-out test set, which was not used for checkpoint selection, hyperparameter tuning, or model configuration. Unless otherwise specified, the quantitative results reported in the comparison and ablation tables were obtained on the held-out test set, whereas the per-epoch performance curves were computed on the validation set to visualize training dynamics. For clarity, absolute differences between performance metrics reported on a 0–1 scale are expressed in percentage points (pp), rather than as relative percentage changes.

To control stochastic variation and ensure fair comparisons, the random seed was fixed at 0 in the original comparative and ablation experiments. To further assess run-to-run variability, additional repeated experiments were conducted for YOLOv8n and Structure-Driven YOLOv8n using three independent random seeds (0, 42, and 1020), while keeping the dataset partition, training settings, checkpoint selection criterion, and evaluation protocol unchanged.

## 3. Results and Discussion

### 3.1. Comparison with Existing Architectures

To validate the effectiveness of the proposed Structure-Driven YOLOv8n, comparative experiments were conducted against several representative object detection architectures, including Faster R-CNN with ResNet-50-FPN backbone, YOLOv5n, YOLOv7-tiny, YOLOv8n, YOLOv10n, YOLOv11n, and YOLOv12n. All models were trained and evaluated using identical dataset partitions and a unified evaluation protocol to ensure fairness and comparability. For each model, the checkpoint corresponding to the highest validation mAP@0.5 was selected and subsequently evaluated on the held-out test set. The evaluation metrics included Precision, Recall, mAP@0.5, and mAP@0.5:0.95. Model complexity was additionally assessed using the parameter count and GFLOPs. The parameter counts were obtained from the corresponding model architectures, whereas GFLOPs were calculated using an input resolution of 640×640. Accordingly, all detection-performance results reported in Table 1 were obtained on the held-out test set.

As shown in Table 1, the compared detectors exhibit noticeable performance differences on the held-out test set. As a representative two-stage detector, Faster R-CNN with ResNet-50-FPN backbone achieves an mAP@0.5 of 0.6120, an mAP@0.5:0.95 of 0.2321, a Precision of 0.7162, and a Recall of 0.5429. Although it achieves competitive detection performance, it requires 41.35 M parameters and 131.4 GFLOPs, which are considerably higher than those of the evaluated lightweight YOLO architectures. Among the YOLO baselines, YOLOv8n demonstrates strong and well-balanced overall performance, achieving the highest mAP@0.5 of 0.6332 while remaining competitive in mAP@0.5:0.95, Precision, and Recall. Notably, YOLOv11n achieves a marginally higher mAP@0.5:0.95, whereas YOLOv12n obtains a higher Recall, indicating that no single baseline architecture dominates all evaluation metrics. Compared with earlier versions such as YOLOv5n and YOLOv7-tiny, YOLOv8n shows stronger overall detection performance on the held-out test set. The results in Table 1 were used only for final independent evaluation and did not participate in baseline architecture or checkpoint selection. The complexity comparison further shows that the evaluated YOLO architectures maintain relatively compact parameter counts and computational requirements compared with Faster R-CNN.

It should be emphasized that the purpose of the YOLO-based comparison was not merely to determine which of YOLOv5n, YOLOv7-tiny, YOLOv8n, YOLOv10n, YOLOv11n, and YOLOv12n achieved the best detection performance. Rather, the primary objective of this study was to validate the effectiveness of the proposed Structure-Driven Feature Modeling method. Following the validation-based comparison using the highest validation mAP@0.5 as the selection criterion, YOLOv8n was adopted as the primary platform for method development and systematic validation. Its architectural maturity, optimization stability, and module extensibility, as discussed in Section 2.2, provide additional practical justification for this role but do not constitute alternative model-selection criteria. To further investigate whether the proposed method depended on this specific architecture, the same structure-driven enhancement mechanism was subsequently integrated into YOLOv5n, YOLOv7-tiny, YOLOv10n, YOLOv11n, and YOLOv12n. As reported in the cross-architecture experiments, the consistent improvements across the evaluated models indicate that the proposed method is not limited to YOLOv8n and support its transferability within the evaluated YOLO family.

By incorporating the proposed Structure-Driven Feature Enhancement Network into YOLOv8n, the detection performance is further improved. Specifically, Structure-Driven YOLOv8n achieves an mAP@0.5 of 0.6647 and an mAP@0.5:0.95 of 0.2691, corresponding to improvements of 3.15 pp and 1.96 pp over the original YOLOv8n, respectively. Meanwhile, the Precision increases from 0.7486 to 0.8241, indicating an improvement in the reliability of the detection results. Structure-Driven YOLOv8n contains 3.14 M parameters and requires 9.8 GFLOPs, compared with 3.01 M parameters and 8.2 GFLOPs for the original YOLOv8n. Therefore, the proposed enhancement mechanism introduces an additional 0.13 M parameters and 1.6 GFLOPs while improving all four detection metrics. These results indicate that the proposed method strengthens the representation of boundary, contour, and morphological cues, thereby improving localization accuracy and overall detection performance under the evaluated conditions.

To further assess the stability of the observed performance gains against run-to-run variation, additional repeated experiments were conducted for YOLOv8n and Structure-Driven YOLOv8n using three independent random seeds (0, 42, and 1020). The dataset partition, training settings, checkpoint selection criterion, and evaluation protocol were kept unchanged across all runs. For each random seed, the checkpoint corresponding to the highest validation mAP@0.5 was selected and subsequently evaluated on the held-out test set. The results of the individual runs, together with the mean and standard deviation across the three independent runs, are reported in Table 2.

As shown in Table 2, Structure-Driven YOLOv8n consistently outperforms the original YOLOv8n across all three random seeds. The mean mAP@0.5 increases from 0.6322±0.0010 to 0.6646±0.0006, while the mean mAP@0.5:0.95 improves from 0.2505±0.0009 to 0.2688±0.0009. Precision and Recall also increase from 0.7483±0.0003 and 0.5548±0.0004 to 0.8240±0.0011 and 0.5935±0.0007, respectively. Moreover, the relatively small standard deviations and the improvements observed across all three seeds indicate that the performance gains remain stable across the evaluated random initializations rather than being attributable to a single training run.

**Figure 8:**
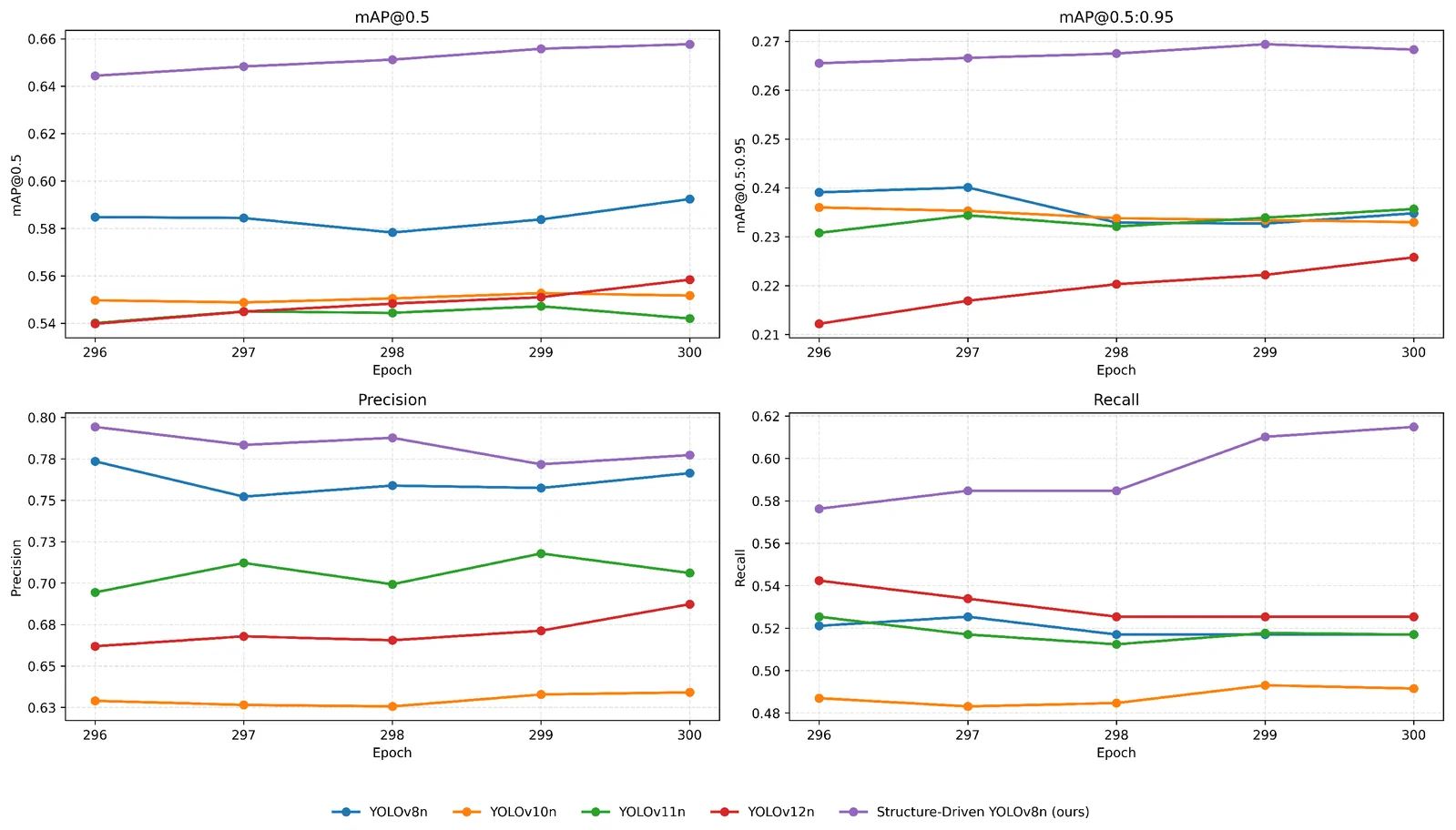
Validation performance curves of different YOLO architectures over the final five training epochs.

To further analyze the late-stage performance stability of several recent YOLO architectures and provide complementary evidence for the use of YOLOv8n as the primary development baseline, an additional validation set analysis was conducted. Specifically, the validation performance of YOLOv8n, YOLOv10n, YOLOv11n, YOLOv12n, and Structure-Driven YOLOv8n over the final five training epochs was recorded, including mAP@0.5, mAP@0.5:0.95, Precision, and Recall. The quantitative results are presented in Table 3. Furthermore, to provide a more intuitive comparison of the late-stage performance behavior of these models, the corresponding validation curves are illustrated in Figure 8.

This analysis is intended as a complementary assessment of late-stage validation stability rather than as an alternative checkpoint selection procedure, and it did not alter the unified validation-based protocol described in Section 2.7. As shown in Table 3 and Figure 8, YOLOv8n maintains stronger and relatively stable validation performance than YOLOv10n, YOLOv11n, and YOLOv12n during the final five epochs, particularly in terms of mAP@0.5 and mAP@0.5:0.95, while Precision and Recall also remain stable. Structure-Driven YOLOv8n further preserves a consistent advantage over the original YOLOv8n. Specifically, its validation mAP@0.5 remains within 0.6444–0.6577, while mAP@0.5:0.95 ranges from 0.2655 to 0.2694. These observations indicate that the selection of YOLOv8n was not dependent on an isolated validation peak and provide complementary evidence for the stability of the proposed enhancement method.

### 3.2. Ablation Study of the Structure-Aware Modules

To further validate the contribution of each module within the proposed Structure-Driven Feature Modeling method to detection performance, systematic ablation studies are conducted on the YOLOv8n baseline. All experiments are performed under identical training settings and experimental conditions to ensure fairness and comparability. By progressively introducing different structure-aware modules, the role of each component in structural feature modeling and its impact on detection performance can be comprehensively analyzed.

This section investigates the contributions from several perspectives. First, the effect of Structure Channel Attention on feature representation is evaluated. Second, the role of Spatial Attention without structural guidance is analyzed. Third, the effectiveness of Structure-Guided Spatial Attention is examined by incorporating both Single-Scale Structure Difference and Multi-Scale Structure Difference. Subsequently, the contribution of the Structure Normalization Module in multi-scale structural feature fusion is assessed. Finally, a comprehensive evaluation is conducted through combined experiments to analyze the overall performance gains brought by the collaborative interaction of all modules.

#### 3.2.1. Ablation Study 1: Effect of Structure Channel Attention

To evaluate the effectiveness of the Structure Channel Attention (SCA) module in structural feature modeling, this study first incorporates the module into the YOLOv8n baseline and conducts comparative experiments against the original model. The SCA module models structural correlations across feature channels, enabling the network to adaptively reweight channel importance. As a result, channels containing salient structural information are emphasized, thereby enhancing the representation of target structural features.

As shown in Table 4, the introduction of Structure Channel Attention leads to consistent improvements across multiple evaluation metrics. Specifically, mAP@0.5 and mAP@0.5:0.95 increase by 0.59 pp and 0.79 pp, respectively, while Precision improves by 4.65 pp and Recall by 0.40 pp. These results demonstrate that the SCA module enhances the network’s focus on key structural feature channels, thereby improving overall detection performance.

In underwater fish detection scenarios, fish targets typically exhibit distinctive morphological structures, such as contours and body shapes. The SCA module highlights channels related to such structural characteristics while suppressing interference from complex underwater backgrounds, thereby improving the model’s ability to perceive discriminative structural information.

#### 3.2.2. Ablation Study 2: Effect of Spatial Attention Without Structural Guidance

To evaluate the role of the Spatial Attention (SA) module in spatial feature modeling, this study incorporates the module into the YOLOv8n baseline and conducts comparative experiments with the original model. The SA module learns importance weights across spatial locations in the feature map, enabling the network to focus on regions where targets are more likely to appear, thereby enhancing feature representation in the spatial domain.

As shown in Table 5, the introduction of Spatial Attention without structural guidance produces consistent improvements across multiple evaluation metrics. Specifically, mAP@0.5 and mAP@0.5:0.95 increase by 0.45 pp and 0.94 pp, respectively, while Precision improves by 3.29 pp and Recall by 0.41 pp. These results indicate that the spatial attention mechanism enhances the network’s responsiveness to target regions, leading to improved detection performance.

In underwater fish detection scenarios, the environment is often characterized by complex backgrounds with abundant irrelevant textures and noise. The Spatial Attention module highlights potential target regions in the spatial domain while suppressing background interference, thereby improving the model’s ability to localize targets and alleviating confusion caused by appearance similarity. However, since this module does not explicitly incorporate structural information, its capability to model structural characteristics remains limited. Therefore, it is necessary to further introduce structure-guided mechanisms to enhance the structural awareness of spatial attention.

#### 3.2.3. Ablation Study 3: Effects of Structure-Guided Spatial Attention, Single-Scale Structure Difference, and Multi-Scale Structure Difference

To further investigate the role of structural information in spatial attention modeling, the Structure-Guided Spatial Attention (SGSA) mechanism is incorporated into the YOLOv8n baseline. Two variants are constructed for comparison: Single-Scale Structure Difference (SSD) and Multi-Scale Structure Difference (MSD). By leveraging structural discrepancy information within feature maps, this module guides spatial attention to focus on regions with prominent structural variations, thereby enhancing the representation of target structural features.

As shown in Table 6, introducing Single-Scale Structure Difference leads to notable performance improvements, with mAP@0.5 and mAP@0.5:0.95 increasing by 1.00 pp and 1.09 pp, respectively, while Precision improves by 5.36 pp and Recall by 0.41 pp. When Multi-Scale Structure Difference is further adopted, the performance is enhanced more substantially: mAP@0.5 and mAP@0.5:0.95 increase by 2.56 pp and 1.32 pp, respectively, with Precision and Recall improving by 6.51 pp and 2.95 pp. These results demonstrate that structure-guided spatial attention effectively strengthens the network’s focus on structurally salient regions, while multi-scale structural modeling further improves the representation of structural features across different scales.

In underwater fish detection scenarios, targets typically exhibit clear structural boundaries and morphological variations, with significant scale differences across images. Single-scale structural modeling captures local structural cues to a certain extent, whereas multi-scale structural differences provide a more comprehensive description of structural variations across scales. This enables more effective discrimination between targets and complex backgrounds and mitigates the impact of blur. Therefore, incorporating Multi-Scale Structure Difference improves detection performance for underwater fish targets.

#### 3.2.4. Ablation Study 4: Effect of Structure Normalization Module

To further evaluate the role of the Structure Normalization Module (SNM) in structural feature modeling, the module is incorporated on top of the Multi-Scale Structure Difference (MSD) and compared with the counterpart without SNM. By applying normalization-based modulation to structural features, SNM stabilizes responses across different scales, thereby improving the consistency of structural representation in the feature space.

As shown in Table 7, the introduction of the Structure Normalization Module leads to modest performance gains. Specifically, mAP@0.5 and mAP@0.5:0.95 increase by 0.13 pp and 0.31 pp, respectively, while Precision improves by 0.43 pp and Recall by 0.17 pp. Since this ablation comparison was conducted using a single random seed, the 0.13 pp improvement in mAP@0.5 is relatively close to the run-to-run variation quantified in Table 2 and should therefore be interpreted cautiously. In contrast, the 0.31 pp improvement in mAP@0.5:0.95 is more clearly separated from the measured variation. Overall, these results support a modest contribution of SNM to detection performance, particularly in terms of mAP@0.5:0.95.

In underwater fish detection scenarios, the complexity of the aquatic environment often leads to instability in structural responses across different scales. By normalizing structural features, SNM enables more stable utilization of multi-scale structural information, thereby further improving detection performance, particularly under partial occlusion conditions.

#### 3.2.5. Ablation Study 5: Combined Effects of Proposed Modules

To further validate the synergistic effects among different structural modules, a series of combinational ablation experiments are conducted on the YOLOv8n baseline by progressively introducing Structure Channel Attention (SCA), Spatial Attention (SA), Multi-Scale Structure Difference (MSD), and the Structure Normalization Module (SNM). By incrementally stacking these modules, the individual contributions and their collaborative effects on overall detection performance can be more clearly analyzed.

For each ablation configuration, the checkpoint corresponding to the highest validation mAP@0.5 was selected and subsequently evaluated on the held-out test set. Accordingly, all quantitative results reported in Table 8 were obtained on the held-out test set. As shown in Table 8, the model performance improves consistently with the progressive integration of structural modules. When SCA and SA are sequentially added to the baseline, mAP@0.5 and mAP@0.5:0.95 increase by 0.94 pp and 1.14 pp, respectively, while Precision and Recall improve by 5.21 pp and 1.06 pp. Building upon this configuration, the introduction of Multi-Scale Structure Difference (MSD) yields gains, with mAP@0.5 and mAP@0.5:0.95 increasing by 2.61 pp and 1.51 pp, respectively, and Precision and Recall improving by 6.78 pp and 3.09 pp. Finally, incorporating the Structure Normalization Module (SNM) achieves the best performance, with mAP@0.5 and mAP@0.5:0.95 improving by 3.15 pp and 1.96 pp, respectively, while Precision and Recall increase by 7.55 pp and 3.80 pp.

These results demonstrate strong complementarity among the proposed modules. SCA and SA enhance feature representation in the channel and spatial domains, respectively, while MSD further strengthens structural feature representation through multi-scale structural difference modeling. SNM subsequently stabilizes multi-scale structural responses via normalization. Through their collaborative interaction, the proposed Structure-Driven Feature Modeling method effectively captures structural information, leading to performance improvements in complex underwater detection scenarios.

### 3.3. Ablation Study on Structure Enhancement at Different Feature Levels

To further analyze the effectiveness of the proposed Structure-Driven Feature Enhancement Network across different feature levels, we conduct comparative experiments by applying the network to the P3, P4, and P5 layers of the YOLOv8 detection head. These feature levels correspond to different representation hierarchies: P3 preserves rich spatial details due to its high resolution, whereas P4 and P5 progressively encode stronger semantic information at the cost of reduced structural details.

All quantitative results reported in Table 9 were obtained on the held-out test set. As shown in Table 9, introducing the proposed network only at the P3 layer yields the most significant performance improvement, with mAP@0.5 and mAP@0.5:0.95 increasing by 3.15 pp and 1.96 pp, respectively, while Precision and Recall improve by 7.55 pp and 3.80 pp. This indicates that performing structural enhancement at high-resolution feature levels is more effective for capturing object boundaries and morphological structures. Moreover, in underwater fish detection scenarios, targets are often small to medium in size; such objects exhibit clearer spatial structures in high-resolution feature maps, making P3 particularly suitable for structural enhancement.

In contrast, introducing the network only at P4 or P5 results in relatively limited performance gains. Among them, P5 achieves slightly better detection performance than P4. This can be attributed to the fact that P4 lies at an intermediate level, lacking both the rich spatial details of P3 and the strong semantic representation of P5, thus limiting the effectiveness of the structure-driven mechanism. Although P5 suffers from reduced structural details due to repeated downsampling, its richer high-level semantics still contribute to target discrimination, resulting in slightly better performance than P4.

Furthermore, applying the proposed network simultaneously to multiple feature levels does not lead to additional performance improvements. This suggests that structural enhancement at the P3 level is sufficient to capture key structural information of targets, while redundant enhancement across multiple scales may introduce feature redundancy, thereby affecting the stability of feature representations.

**Figure 9:**
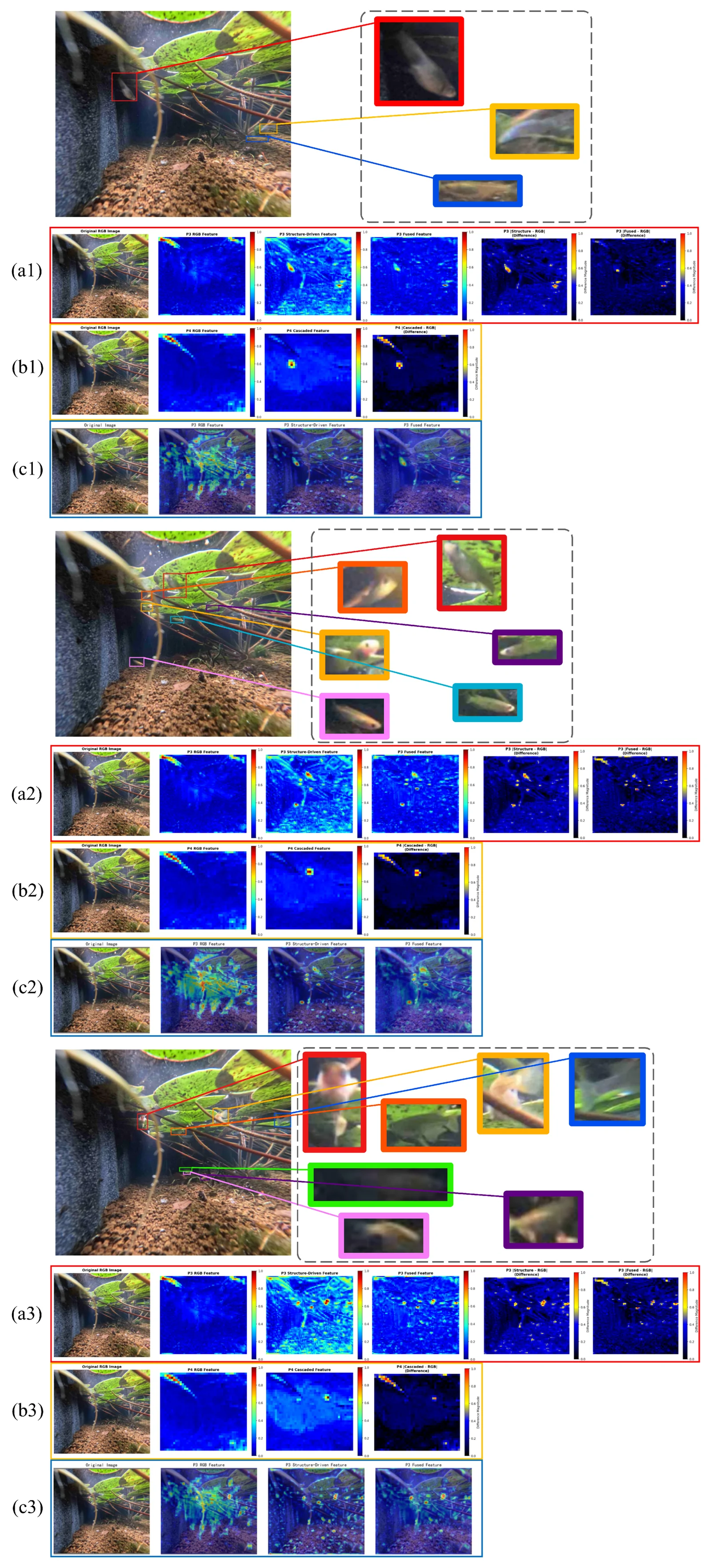
Visualization of channel-mean activation maps and Grad-CAM heatmaps at the P3 and P4 layers. (**a1**–**a3**) P3 channel-mean activation maps. From left to right: Original RGB image, P3 RGB feature, P3 structure-driven feature, P3 fused feature, P3 $|F_{\mathrm{structure}}-F_{\mathrm{rgb}}|$, P3 $|F_{\mathrm{fused}}-F_{\mathrm{rgb}}|$. (**b1**–**b3**) P4 channel-mean activation maps. From left to right: Original RGB image, P4 RGB feature, P4 cascaded feature, P4 $|F_{\mathrm{cascaded}}-F_{\mathrm{rgb}}|$. (**c1**–**c3**) P3 Grad-CAM heatmaps. From left to right: Original image, P3 RGB feature, P3 structure-driven feature, P3 fused feature.

To further analyze the effect of the proposed structure-driven feature enhancement method, we focus on its application at the P3 level, where low- and mid-level structural information is most prominent. As illustrated in Figure 9, we compare the original P3 features, structure-enhanced features, and their fused representations. In addition, feature responses at the P4 level are examined to evaluate the cross-layer propagation of structural information from P3. Specifically, channel-mean activation maps and corresponding difference maps are visualized at both P3 and P4 levels to characterize the impact of structural enhancement and its transmission across feature hierarchies. Furthermore, Grad-CAM is employed at the P3 level to highlight the discriminative regions that directly influence the detection results.

The results demonstrate that the proposed method enhances structural responses in low-level features and facilitates their stable propagation to higher-level representations, thereby improving overall feature expressiveness. At the P3 level, structure-enhanced features exhibit a stronger ability to localize target regions based on structural cues. However, they may also introduce certain environmental noise. By fusing structural features with the original RGB features, the model is able to emphasize target regions while suppressing background interference. In contrast, the original P3 RGB features tend to focus on background elements such as aquatic vegetation, failing to capture key target information. The difference maps further confirm that the extracted discrepancies correspond closely to the regions of interest, indicating that the structural enhancement successfully isolates discriminative features. At the P4 level, the original RGB features are largely dominated by background noise, whereas the propagated features from the enhanced P3 representation are able to capture more salient object structures, particularly for larger targets. Nevertheless, small objects remain partially suppressed due to the loss of fine-grained details during downsampling.

The Grad-CAM visualizations provide additional evidence of the effectiveness of the proposed method. The baseline P3 RGB features primarily respond to background noise, while the structure-enhanced features suppress irrelevant regions and highlight target locations based on structural cues. The fused features further improve this behavior by maintaining strong target responses while reducing environmental interference.

For visualization, the feature tensor at each level is first averaged along the channel dimension to obtain a two-dimensional activation map M(x,y). The map is then normalized to [0,1] using min–max normalization and visualized using a jet colormap, where cooler colors (e.g., blue to cyan) indicate lower activation values and warmer colors (e.g., yellow to red) indicate higher responses. It should be noted that the color mapping is only meaningful within each individual image and does not correspond to absolute physical intensity.

Difference maps are computed as the pixel-wise absolute differences between channel-mean features, including $\left|F_{\mathrm{structure}}-F_{\mathrm{rgb}}\right|$ and $\left|F_{\mathrm{fused}}-F_{\mathrm{rgb}}\right|$. Each map is normalized to [0,1] based on its global maximum and visualized using a custom colormap ranging from black through blue to yellow–orange–red. Dark regions indicate minor differences, whereas bright warm colors highlight areas with significant deviations introduced by structural enhancement or feature fusion.

In addition, Grad-CAM is employed to visualize discriminative regions for detection. Specifically, gradients of the target class score are backpropagated to the selected feature layer to produce a coarse spatial response map, followed by ReLU activation, upsampling to the input resolution, and min–max normalization. The resulting saliency map is overlaid on the RGB image using a pseudocolor scheme, where red–yellow regions indicate strong contributions to the prediction and blue regions indicate weak contributions.

All visualizations are normalized on a per-image basis; therefore, color intensity is intended solely for qualitative analysis within a single image and should not be used for quantitative comparison across different images or feature levels.

### 3.4. Controlled Evaluation of the Cross-Architecture Transferability and Effectiveness of the Structure-Driven Feature Modeling Mechanism

The purpose of this experiment is not to establish a comprehensive performance ranking against all existing underwater- or fish-specific detection systems, but to examine whether the proposed Structure-Driven Feature Modeling mechanism can produce consistent improvements when applied to different YOLO architectures. Accordingly, controlled paired comparisons were conducted using YOLOv5n, YOLOv7-tiny, YOLOv8n, YOLOv10n, YOLOv11n, and YOLOv12n together with their corresponding structure-driven variants. In all enhanced models, the proposed mechanism was incorporated at the P3 level.

For each original model and its structure-driven counterpart, the dataset partition, training strategy, experimental settings, checkpoint selection criterion, and held-out test evaluation protocol were kept unchanged. For each model, the checkpoint corresponding to the highest validation mAP@0.5 was selected independently and subsequently evaluated on the held-out test set. Accordingly, all quantitative results reported in Table 10 were obtained on the held-out test set. Since the principal controlled difference within each paired comparison was the inclusion of the proposed Structure-Driven Feature Modeling mechanism, the observed performance changes can be more directly attributed to the proposed mechanism rather than to differences in dataset composition, training strategy, checkpoint selection, or evaluation protocol.

Accordingly, in this section, cross-architecture transferability refers specifically to the ability of the same Structure-Driven Feature Modeling mechanism to improve the tested YOLO architectures under the Medaka dataset and unified experimental protocol. This architectural transferability is distinct from the cross-environment effectiveness examined in Section 3.5, where YOLOv8n and Structure-Driven YOLOv8n were additionally evaluated on the independent public Blue Bot Dataset containing different fish species and underwater scene conditions.

As shown in Table 10, integrating the Structure-Driven Network consistently improves performance across all architectures to varying degrees. For YOLOv5n, mAP@0.5 and mAP@0.5:0.95 increase by 2.53 pp and 1.84 pp, respectively, while Precision and Recall improve by 7.15 pp and 6.24 pp. For YOLOv7-tiny, the corresponding improvements are 4.23 pp and 1.88 pp in mAP@0.5 and mAP@0.5:0.95, together with gains of 7.39 pp in Precision and 7.83 pp in Recall. For YOLOv8n, mAP@0.5 and mAP@0.5:0.95 increase by 3.15 pp and 1.96 pp, respectively, while Precision and Recall improve by 7.55 pp and 3.80 pp. For YOLOv10n, the improvements are 4.35 pp and 0.97 pp in mAP@0.5 and mAP@0.5:0.95, with Precision and Recall increasing by 7.28 pp and 7.74 pp. In YOLOv11n, mAP@0.5 and mAP@0.5:0.95 improve by 1.32 pp and 1.56 pp, accompanied by gains of 8.26 pp in Precision and 3.30 pp in Recall. Similarly, for YOLOv12n, the proposed method yields improvements of 2.14 pp and 1.65 pp in mAP@0.5 and mAP@0.5:0.95, respectively, along with increases of 8.19 pp in Precision and 2.15 pp in Recall.

Overall, the proposed Structure-Driven Feature Modeling mechanism consistently improves detection performance across all six evaluated YOLO architectures, with particularly notable improvements in Precision. Because the experimental settings were kept unchanged between each original architecture and its corresponding enhanced version, the observed performance differences within the paired comparisons can be more directly attributed to the proposed mechanism. The consistent gains across YOLOv5n, YOLOv7-tiny, YOLOv8n, YOLOv10n, YOLOv11n, and YOLOv12n further indicate that the observed effect is not specific to YOLOv8n and support the cross-architecture transferability of the proposed method within the evaluated YOLO family.

However, because a separately reimplemented and tuned underwater- or fish-specific detector was not included under the same Medaka dataset partition and evaluation protocol, the present results do not support a claim of superiority over all existing domain-specific detection systems. Direct evaluation against such uniformly trained and optimized competitors remains an important direction for future work.

**Figure 10:**
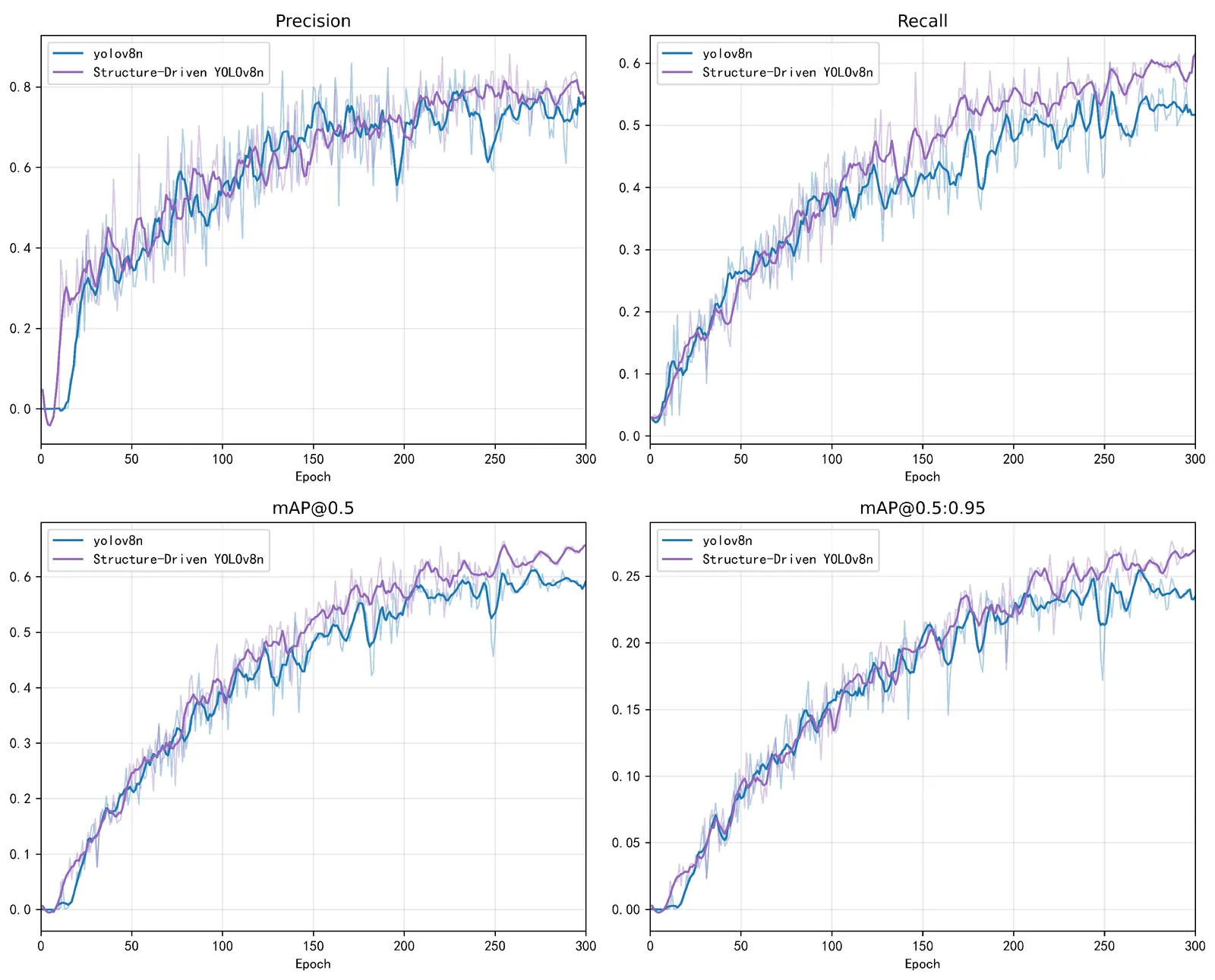
Validation performance comparison of YOLOv8n and Structure-Driven YOLOv8n over 300 training epochs.

**Figure 11:**
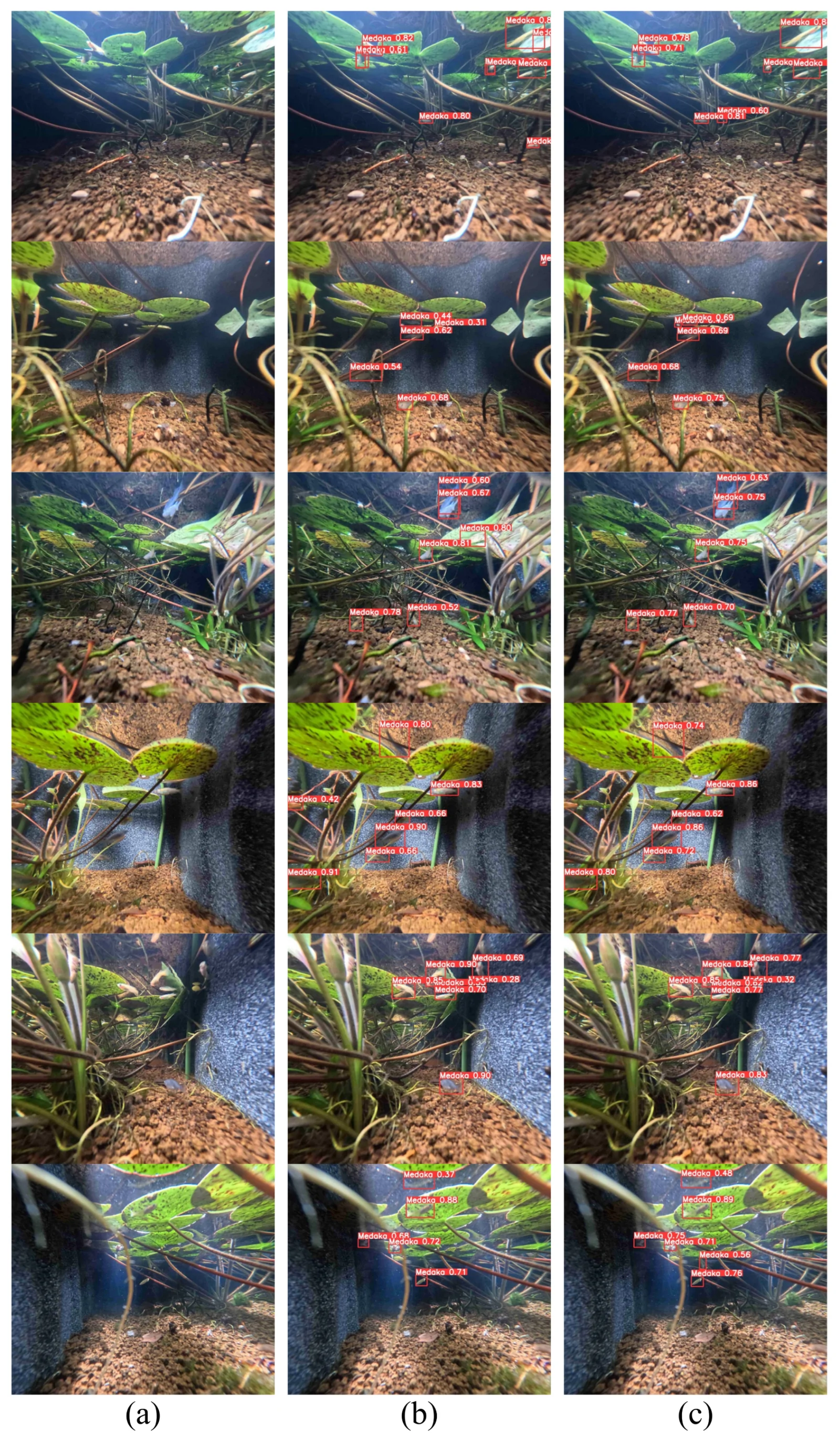
Comparison of detection results between YOLOv8n and Structure-Driven YOLOv8n. (**a**) Original image, (**b**) YOLOv8n, (**c**) Structure-Driven YOLOv8n.

To provide a more intuitive visualization of the training dynamics, Figure 10 presents the validation set performance curves of YOLOv8n and Structure-Driven YOLOv8n on the Medaka dataset over 300 training epochs. The raw per-epoch curves naturally exhibit short-term fluctuations during an individual training run. As the models gradually approach convergence, Structure-Driven YOLOv8n generally exhibits a higher overall validation performance trend than the original YOLOv8n during the later stages of training. For visualization purposes only, the curves are smoothed using the Savitzky–Golay filter to suppress short-term local fluctuations and make the overall performance trends easier to observe. The original unsmoothed validation values are retained as light-colored curves for reference. The smoothed curves were not used for checkpoint selection, quantitative performance calculation, or statistical comparison; all such analyses were based on the original unsmoothed metric values. Figure 11 further illustrates qualitative detection results, showing that the proposed method achieves more accurate localization and more stable predictions in complex underwater environments, with particularly notable improvements for small-scale objects, occluded targets, blurred boundaries, and visually similar instances.

**Figure 12:**
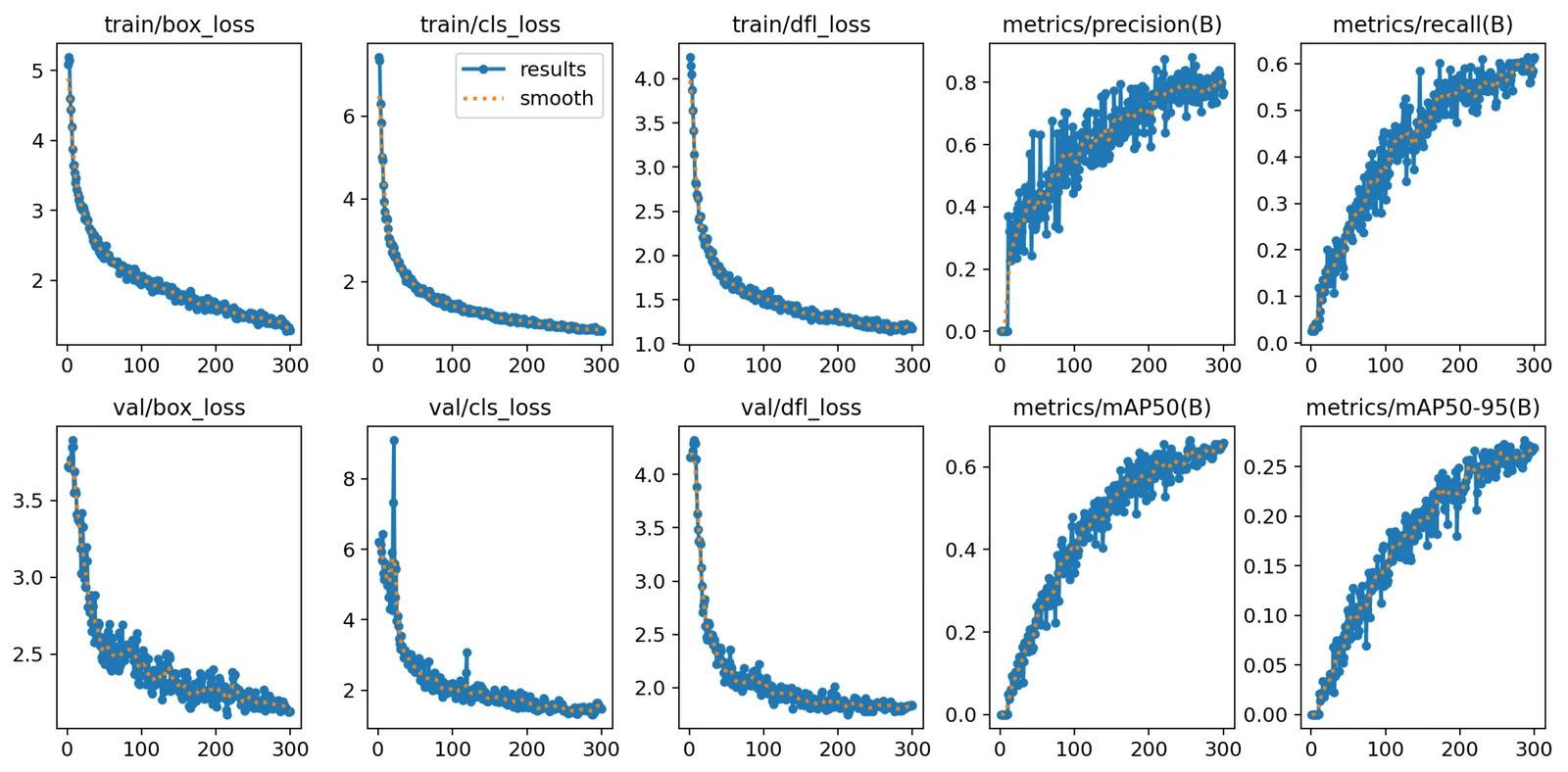
Training and validation performance curves of Structure-Driven YOLOv8n over 300 epochs.

Furthermore, to analyze the convergence behavior of the proposed model, Figure 12 depicts the training and validation curves of Structure-Driven YOLOv8n over 300 epochs, including various loss components as well as mAP@0.5, mAP@0.5:0.95, Precision, and Recall. The results indicate that, after 300 epochs, all loss terms converge smoothly, and no evident overfitting is observed on the validation set, demonstrating both stable convergence and reliable detection performance on the dataset.

### 3.5. External Validation on a Public Underwater Fish Dataset

**Figure 13:**
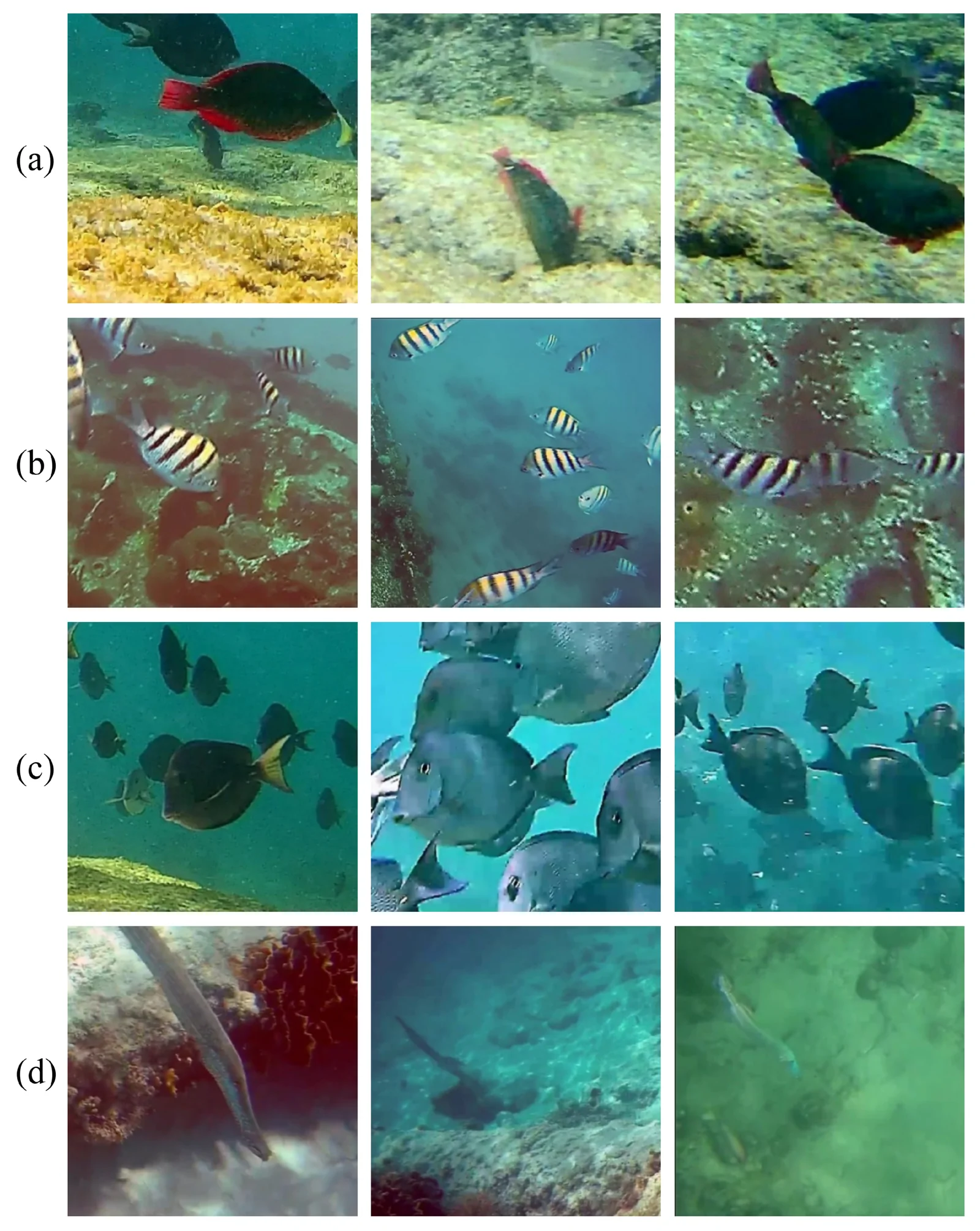
Representative samples from the Blue Bot Dataset across diverse underwater scenes: (**a**) Parrotfish, (**b**) Sergeant Major fish, (**c**) Atlantic Tang, and (**d**) Trumpetfish.

To further evaluate whether the effectiveness of the proposed Structure-Driven Feature Modeling mechanism extends beyond the Medaka tank environment, an additional external validation experiment was conducted on the publicly available Blue Bot Dataset. The dataset contains four marine fish species, including Parrotfish, Sergeant Major fish, Atlantic Tang, and Trumpetfish, as illustrated in Figure 13. The public Blue Bot Dataset contains a total of 2409 underwater images and covers several challenging conditions commonly encountered in underwater fish detection, including partial occlusion, target–background similarity, blurred target appearance, and locally dense fish distributions.

Under the same experimental settings and evaluation protocol described in Section 2.7, the original YOLOv8n and the proposed Structure-Driven YOLOv8n were trained and evaluated using the predefined dataset partition. For both models, checkpoint selection was performed exclusively according to the highest validation mAP@0.5, and the selected checkpoints were subsequently evaluated on the held-out test subset. This controlled comparison was conducted to examine whether the proposed Structure-Driven Feature Modeling mechanism remains effective on an external underwater dataset involving fish species, background compositions, image characteristics, and scene conditions that differ from those represented in the Medaka dataset. The quantitative results are presented in Table 11.

As shown in Table 11, Structure-Driven YOLOv8n consistently outperforms the original YOLOv8n across all four overall evaluation metrics on the Blue Bot Dataset. Specifically, the overall mAP@0.5 increases from 0.7671 to 0.8007, corresponding to an improvement of 3.36 pp, while mAP@0.5:0.95 increases from 0.4026 to 0.4211, representing an improvement of 1.85 pp. Precision and Recall are also improved from 0.7588 and 0.6921 to 0.8073 and 0.7427, corresponding to gains of 4.85 pp and 5.06 pp, respectively. These improvements indicate that the proposed method enhances both the reliability of positive predictions and the ability to detect fish instances in the external underwater dataset.

At the category level, consistent improvements are observed across all four fish species. For Parrotfish, mAP@0.5 increases from 0.6512 to 0.7174, while mAP@0.5:0.95 increases from 0.3212 to 0.3502, corresponding to improvements of 6.62 pp and 2.90 pp, respectively. Precision and Recall also increase by 5.78 pp and 6.43 pp. These gains indicate that the proposed method provides more reliable feature representations for Parrotfish, which exhibit relatively lower baseline performance than the other evaluated categories.

For Sergeant Major fish, the original YOLOv8n already achieves relatively high detection performance. Nevertheless, Structure-Driven YOLOv8n further improves mAP@0.5 from 0.8589 to 0.8618 and mAP@0.5:0.95 from 0.4260 to 0.4307. Precision and Recall increase from 0.7695 and 0.8466 to 0.7882 and 0.8636, corresponding to gains of 1.87 pp and 1.70 pp, respectively. The improvements under an already strong baseline indicate that the proposed mechanism remains beneficial even for categories with comparatively favorable detection performance.

For Atlantic Tang, Structure-Driven YOLOv8n improves mAP@0.5 from 0.7953 to 0.8530 and mAP@0.5:0.95 from 0.4516 to 0.4804, corresponding to gains of 5.77 pp and 2.88 pp, respectively. Precision increases from 0.7488 to 0.7635, while Recall increases from 0.7380 to 0.7857. The simultaneous improvement in detection accuracy and recall suggests that the structure-driven mechanism strengthens the representation of target contours and morphological information under varying underwater backgrounds.

For Trumpetfish, mAP@0.5 and mAP@0.5:0.95 increase from 0.7628 and 0.4115 to 0.7706 and 0.4231, corresponding to improvements of 0.78 pp and 1.16 pp, respectively. More noticeable improvements are observed in Precision and Recall, which increase from 0.7478 and 0.6005 to 0.8507 and 0.6737, corresponding to gains of 10.29 pp and 7.32 pp. These results indicate that the proposed method substantially reduces false-positive detections while improving the detection of missed Trumpetfish instances.

Overall, the proposed Structure-Driven YOLOv8n achieves improvements across all evaluated metrics and all four fish species on the Blue Bot Dataset. Unlike the Medaka dataset, the Blue Bot Dataset contains different fish species, image sources, underwater backgrounds, and scene compositions. The consistent improvements obtained on this independent public dataset provide external experimental evidence that the effectiveness of the proposed Structure-Driven Feature Modeling mechanism is not restricted to the self-collected Medaka dataset. Together with the results obtained on the Medaka dataset, these findings support the cross-environment effectiveness of structure-driven feature learning for underwater fish detection.

## 4. Conclusions

In this paper, we address the challenge of fish detection under complex underwater conditions by developing a structure-enhanced detection method, termed Structure-Driven YOLOv8n. By introducing a structure-aware feature modeling mechanism into the high-resolution P3 branch of the feature pyramid, the proposed approach enhances feature representations in both channel and spatial dimensions, thereby strengthening the representation of structural information. Specifically, the proposed mechanism coordinates several functional components, including Structure Channel Attention, Multi-Scale Structure Difference, Structure Normalization Module, and Structure-Guided Spatial Attention, and further combines a Structure-Driven Feature Modeling method with a Structure-Driven Fusion strategy to model multi-scale structural cues while retaining complementary appearance information.

Extensive controlled experiments on the self-collected Medaka dataset demonstrate that the proposed Structure-Driven Feature Modeling mechanism improves mAP@0.5, mAP@0.5:0.95, Precision, and Recall when integrated into the evaluated YOLO architectures. The ablation studies verify the contribution of the individual structure-aware components, while the feature-level analysis indicates that structural enhancement is particularly effective at the high-resolution P3 feature level. Moreover, controlled paired comparisons involving YOLOv5n, YOLOv7-tiny, YOLOv8n, YOLOv10n, YOLOv11n, and YOLOv12n show consistent improvements over their corresponding original baselines, supporting the effectiveness and cross-architecture transferability of the proposed mechanism within the evaluated YOLO family.

Additional external validation on the public Blue Bot Dataset provides further evidence of the effectiveness of the proposed method across different fish species and underwater scene conditions. Compared with the original YOLOv8n, Structure-Driven YOLOv8n improves mAP@0.5 from 0.7671 to 0.8007 and mAP@0.5:0.95 from 0.4026 to 0.4211, while Precision and Recall increase from 0.7588 and 0.6921 to 0.8073 and 0.7427, respectively. These results provide external experimental evidence that the effectiveness of the proposed mechanism is not restricted to the self-collected Medaka tank dataset.

Overall, the proposed method enhances structural responses in shallow features and improves detection performance under the underwater conditions represented in the Medaka and Blue Bot datasets, including scenes involving small targets, blurred boundaries, partial occlusion, and visually similar targets.

The scope of these findings should nevertheless be interpreted within the current experimental setting. First, the present study does not include a separately reimplemented and tuned underwater- or fish-specific detector evaluated under the same Medaka dataset partition, training settings, checkpoint selection criterion, and evaluation protocol. Accordingly, the reported results are not intended to establish superiority over all existing domain-specific underwater or fish detection systems, but rather to demonstrate the effectiveness of the proposed Structure-Driven Feature Modeling mechanism through controlled paired comparisons. Second, although the external evaluation on the Blue Bot Dataset provides evidence of the cross-environment effectiveness of the proposed method across different fish species, underwater backgrounds, and scene conditions, the current evaluation remains limited to the two underwater datasets considered in this study. Further validation across additional aquaculture facilities, natural water bodies, imaging devices, geographically distinct sites, and open-water field conditions is still required. Third, the repeated-run experiments were conducted using a fixed Medaka dataset partition, and variability across independently defined splits that were disjoint by time, camera position, or scene was not evaluated. Therefore, the current statistical analysis characterizes run-to-run training variability but does not quantify split variability. Accordingly, the conclusions of this study are restricted to the effectiveness observed on the Medaka and Blue Bot datasets and the cross-architecture transferability demonstrated on the Medaka dataset within the evaluated YOLO family.

Future work will extend the proposed Structure-Driven Feature Modeling mechanism to additional domain-specific underwater and fish detection frameworks and evaluate them under unified experimental conditions. Further external validation will be conducted using additional multi-site datasets, different imaging devices, geographically distinct aquaculture environments, natural water bodies, and open-water field data to more comprehensively assess environmental and cross-domain generalization. Future studies will also evaluate independently defined data partitions that are disjoint by time, camera position, or scene to quantify split variability and assess scene-level robustness. In addition, future research will explore more lightweight structural designs to reduce computational overhead, jointly model structural and temporal information for video-based detection, and extend the proposed mechanism to related tasks such as object tracking and instance segmentation.

## Figures and Tables

**Table 1:** Comparison of detection performance and computational complexity with representative object detectors.

Model	mAP@0.5	mAP@0.5:0.95	Precision	Recall	Parameters (M)	GFLOPs (G)
Faster R-CNN (ResNet-50-FPN)	0.6120	0.2321	0.7162	0.5429	41.35	131.4
YOLOv5n	0.5948	0.2335	0.7700	0.5107	2.51	7.2
YOLOv7-tiny	0.5421	0.2183	0.6620	0.4723	6.02	12.5
YOLOv8n	0.6332	0.2495	0.7486	0.5552	3.01	8.2
YOLOv10n	0.5550	0.2249	0.6691	0.4830	2.71	8.4
YOLOv11n	0.6176	0.2498	0.7284	0.5517	2.59	6.4
YOLOv12n	0.5985	0.2359	0.6749	0.5678	2.55	6.5
Structure-Driven YOLOv8n (ours)	**0.6647**	**0.2691**	**0.8241**	**0.5932**	**3.14**	**9.8**

**Table 2:** Repeated-run performance comparison of YOLOv8n and Structure-Driven YOLOv8n using three independent random seeds.

Model	Seed	mAP@0.5	mAP@0.5:0.95	Precision	Recall
YOLOv8n	0	0.6332	0.2495	0.7486	0.5552
	42	0.6312	0.2508	0.7480	0.5545
	1020	0.6321	0.2513	0.7482	0.5546
	Mean ± Std	0.6322±0.0010	0.2505±0.0009	0.7483±0.0003	0.5548±0.0004
Structure-Driven YOLOv8n	0	0.6647	0.2691	0.8241	0.5932
	42	0.6640	0.2678	0.8229	0.5930
	1020	0.6651	0.2694	0.8250	0.5943
	Mean ± Std	0.6646±0.0006	0.2688±0.0009	0.8240±0.0011	0.5935±0.0007

**Table 3:** Detection performance of different YOLO architectures during the final five training epochs.

Model	Epoch	mAP@0.5	mAP@0.5:0.95	Precision	Recall
YOLOv8n	296	0.5848	0.2391	0.7735	0.5211
	297	0.5844	0.2401	0.7522	0.5254
	298	0.5783	0.2329	0.7589	0.5170
	299	0.5838	0.2327	0.7574	0.5170
	300	0.5924	0.2348	0.7664	0.5170
	**Avg**	**0.5847**	**0.2359**	**0.7617**	**0.5195**
YOLOv10n	296	0.5497	0.2360	0.6290	0.4870
	297	0.5488	0.2353	0.6265	0.4831
	298	0.5505	0.2338	0.6256	0.4847
	299	0.5527	0.2334	0.6328	0.4931
	300	0.5517	0.2330	0.6341	0.4915
	**Avg**	**0.5507**	**0.2343**	**0.6296**	**0.4879**
YOLOv11n	296	0.5401	0.2308	0.6944	0.5254
	297	0.5450	0.2344	0.7122	0.5170
	298	0.5444	0.2321	0.6993	0.5124
	299	0.5472	0.2339	0.7179	0.5177
	300	0.5420	0.2357	0.7061	0.5170
	**Avg**	**0.5437**	**0.2334**	**0.7060**	**0.5179**
YOLOv12n	296	0.5398	0.2122	0.6619	0.5424
	297	0.5449	0.2169	0.6679	0.5339
	298	0.5483	0.2203	0.6655	0.5254
	299	0.5510	0.2222	0.6713	0.5254
	300	0.5584	0.2258	0.6873	0.5254
	**Avg**	**0.5485**	**0.2195**	**0.6708**	**0.5305**
Structure-Driven YOLOv8n (ours)	296	0.6444	0.2655	0.7943	0.5763
	297	0.6483	0.2666	0.7834	0.5848
	298	0.6512	0.2675	0.7877	0.5848
	299	0.6558	0.2694	0.7717	0.6102
	300	0.6577	0.2683	0.7773	0.6149
	**Avg**	**0.6515**	**0.2675**	**0.7829**	**0.5942**

**Table 4:** Effect of the SCA module on detection performance.

Model	mAP@0.5	mAP@0.5:0.95	Precision	Recall
YOLOv8n	0.6332	0.2495	0.7486	0.5552
YOLOv8n + SCA	0.6391	0.2574	0.7951	0.5592

**Table 5:** Effect of the SA module without structural guidance on detection performance.

Model	mAP@0.5	mAP@0.5:0.95	Precision	Recall
YOLOv8n	0.6332	0.2495	0.7486	0.5552
YOLOv8n + SA	0.6377	0.2589	0.7815	0.5593

**Table 6:** Effects of the SGSA module with SSD and MSD on detection performance.

Model	mAP@0.5	mAP@0.5:0.95	Precision	Recall
YOLOv8n	0.6332	0.2495	0.7486	0.5552
YOLOv8n + SGSA + SSD	0.6432	0.2604	0.8022	0.5593
YOLOv8n + SGSA + MSD	0.6588	0.2627	0.8137	0.5847

**Table 7:** Effect of the SNM module on detection performance.

Model	mAP@0.5	mAP@0.5:0.95	Precision	Recall
MSD	0.6588	0.2627	0.8137	0.5847
MSD + SNM	0.6601	0.2658	0.8180	0.5864

**Table 8:** Combined effects of proposed modules on detection performance.

SCA	SA	MSD	SNM	mAP@0.5	mAP@0.5:0.95	Precision	Recall
				0.6332	0.2495	0.7486	0.5552
✓				0.6391	0.2574	0.7951	0.5592
✓	✓			0.6426	0.2609	0.8007	0.5658
✓	✓	✓		0.6593	0.2646	0.8164	0.5861
✓	✓	✓	✓	0.6647	0.2691	0.8241	0.5932

**Table 9:** Effect of Structure Enhancement at Different Feature Levels on Detection Performance.

P3	P4	P5	mAP@0.5	mAP@0.5:0.95	Precision	Recall
			0.6332	0.2495	0.7486	0.5552
✓			0.6647	0.2691	0.8241	0.5932
	✓		0.5995	0.2644	0.7501	0.5297
		✓	0.6429	0.2578	0.7621	0.5508
✓	✓		0.6587	0.2551	0.7547	0.6017
✓		✓	0.6583	0.2639	0.7737	0.5690
	✓	✓	0.6045	0.2579	0.7542	0.4644
✓	✓	✓	0.6255	0.2462	0.7834	0.5236

**Table 10:** Controlled paired performance comparison between the original YOLO architectures and their Structure-Driven counterparts on the held-out test set.

Model	mAP@0.5	mAP@0.5:0.95	Precision	Recall
YOLOv5n	0.5948	0.2335	0.7700	0.5107
YOLOv5n + Structure-Driven	0.6201 **(+2.53 pp)**	0.2519 **(+1.84 pp)**	0.8415 **(+7.15 pp)**	0.5731 **(+6.24 pp)**
YOLOv7-tiny	0.5421	0.2183	0.6620	0.4723
YOLOv7-tiny + Structure-Driven	0.5844 **(+4.23 pp)**	0.2371 **(+1.88 pp)**	0.7359 **(+7.39 pp)**	0.5506 **(+7.83 pp)**
YOLOv8n	0.6332	0.2495	0.7486	0.5552
YOLOv8n + Structure-Driven	0.6647 **(+3.15 pp)**	0.2691 **(+1.96 pp)**	0.8241 **(+7.55 pp)**	0.5932 **(+3.80 pp)**
YOLOv10n	0.5550	0.2249	0.6691	0.4830
YOLOv10n + Structure-Driven	0.5985 **(+4.35 pp)**	0.2346 **(+0.97 pp)**	0.7419 **(+7.28 pp)**	0.5604 **(+7.74 pp)**
YOLOv11n	0.6176	0.2498	0.7284	0.5517
YOLOv11n + Structure-Driven	0.6308 **(+1.32 pp)**	0.2654 **(+1.56 pp)**	0.8110 **(+8.26 pp)**	0.5847 **(+3.30 pp)**
YOLOv12n	0.5985	0.2359	0.6749	0.5678
YOLOv12n + Structure-Driven	0.6199 **(+2.14 pp)**	0.2524 **(+1.65 pp)**	0.7568 **(+8.19 pp)**	0.5893 **(+2.15 pp)**

**Table 11:** External validation results of YOLOv8n and Structure-Driven YOLOv8n on the public Blue Bot Dataset.

Model	Class	mAP@0.5	mAP@0.5:0.95	Precision	Recall
YOLOv8n	All	0.7671	0.4026	0.7588	0.6921
	Parrotfish	0.6512	0.3212	0.7691	0.5833
	Sergeant Major fish	0.8589	0.4260	0.7695	0.8466
	Atlantic Tang	0.7953	0.4516	0.7488	0.7380
	Trumpetfish	0.7628	0.4115	0.7478	0.6005
Structure-Driven YOLOv8n	All	0.8007	0.4211	0.8073	0.7427
	Parrotfish	0.7174	0.3502	0.8269	0.6476
	Sergeant Major fish	0.8618	0.4307	0.7882	0.8636
	Atlantic Tang	0.8530	0.4804	0.7635	0.7857
	Trumpetfish	0.7706	0.4231	0.8507	0.6737

## Data Availability

The source code is publicly available at the following GitHub repository: [https://github.com/Kirito-97/Structure-Driven-Feature-Modeling-for-Robust-Fish-Detection-in-Complex-Underwater-Environments] The public Blue Bot Dataset used for external validation is available at: [https://www.kaggle.com/datasets/hiyaro/bluenetpenta]. The Medaka dataset is currently being used in ongoing and planned follow-up studies and will be made publicly available after these studies are completed. The corresponding dataset access information will be provided through the same GitHub repository. During this period, access to the dataset may be requested from the corresponding author upon reasonable request.

## Abbreviations

The following abbreviations are used in this manuscript:

Structure Channel Attention	SCA
Spatial Attention	SA
Structure-Guided Spatial Attention	SGSA
Single-Scale Structure Difference	SSD
Multi-Scale Structure Difference	MSD
Structure Normalization Module	SNM
Percentage Points	pp
Red–Green–Blue	RGB
Average Precision	AP
Average Recall	AR
Mean Average Precision	mAP
Mean Average Recall	mAR
Batch Normalization	BN
False Negative	FN
False Positive	FP
True Positive	TP
Gradient-weighted Class Activation Mapping	Grad-CAM
Intersection over Union	IoU
Rectified Linear Unit	ReLU
Standard Deviation	Std
You Only Look Once	YOLO
Giga Floating-Point Operations	GFLOPs
Feature Pyramid Network	FPN
Residual Network	ResNet
Region-Based Convolutional Neural Network	R-CNN
